# STAT6 inhibits ferroptosis and alleviates acute lung injury via regulating P53/SLC7A11 pathway

**DOI:** 10.1038/s41419-022-04971-x

**Published:** 2022-06-06

**Authors:** Youjing Yang, Yu Ma, Qianmin Li, Yi Ling, Yujia Zhou, Kaimiao Chu, Lian Xue, Shasha Tao

**Affiliations:** 1 Chongqing University Central Hospital&Chongqing Emergency Medical Center, No.1 Jiankang Road, Yuzhong District, Chongqing, 400014 China; 2 School of Public Health, Medical College of Soochow University, 199 Ren’ai Road, Suzhou, 215123 China

**Keywords:** Mechanisms of disease, Respiratory tract diseases

## Abstract

Compelling evidences have revealed the emerging role of ferroptosis in the pathophysiological process of acute lung injury (ALI), but its modulation is not clear. Here, we identified that STAT6 acted as a critical regulator of epithelium ferroptosis during ALI. Firstly, STAT6 expression and activity were increased in the ALI mice models caused by crystalline silica (CS), LPS and X-ray exposure. Followed by confirming the contribution of ferroptosis in the above ALI with ferrostatin-1 and deferoxamine intervention, bioinformatic analyses revealed that STAT6 expression was negatively correlated with ferroptosis. Consistently, lung epithelium-specific depletion of STAT6 in mice or STAT6 knockdown in cultured epithelial cells exacerbated ferroptosis in the above ALI. While overexpression of STAT6 in lung epithelial cells attenuated the ferroptosis. Mechanistically, SLC7A11 is a typical ferroptosis-related gene and negatively regulated by P53. CREB-binding protein (CBP) is a critical acetyltransferase of P53 acetylation, showing valuable regulation on targets’ transcription. Herein, we found that STAT6 negatively regulates ferroptosis through competitively binding with CBP, which inhibits P53 acetylation and transcriptionally restores SLC7A11 expression. Finally, pulmonary-specific STAT6 overexpression decreased the ferroptosis and attenuated CS and LPS induced lung injury. Our findings revealed that STAT6 is a pivotal regulator of ferroptosis, which may be a potential therapeutic target for the treatment of acute lung injury.

## Introduction

Acute lung injury (ALI) is a common clinical syndrome caused by both pulmonary and extra-pulmonary factors, and its most severe form ARDS (acute respiratory syndrome) causes high morbidity and mortality with no effective targeted intervention [1–3]. Previous studies have confirmed that inflammation, coagulation and oxidative stress play important role in the pathogenesis of ALI, which lead to inflammatory cells infiltration, pulmonary edema, arterial hypoxemia, finally results in dysfunction of lung tissue [4, 5]. We also reported that attenuation of oxidative stress and inflammatory response could alleviate the pathological condition of lung injury [6, 7]. Noteworthy, recent studies found that iron mobilization and decompartmentalization have a pathogenic role in both animal models and human cases of ALI, which reveals the contribution of ferroptosis to ALI [8].

Ferroptosis is an iron-dependent new programmed cell death, which is quite different from other classical programmed cell death, including apoptosis, necroptosis, senescence and autophagy both in morphology and biological properties [9, 10]. Hitherto a series studies have revealed that ferroptosis is involved in various diseases, such as tumor, nervous system disease and infection since it was initially proposed by Dixon et al. in 2012 [11–13]. Generally, ferroptosis can be triggered by physiological conditions such as high extracellular glutamate, cystine deprivation, amino acid starvation. It can also be induced through inhibiting system Xc^-^ (Cystine/glutamate transporter), GPX4 (glutathione peroxidase 4). Oppositely, ferroptosis can be suppressed by iron chelators (e.g., deferoxamine) and lipophilic antioxidants (e.g., ferrostatin-1) and inhibition of ferroptosis could diminish related clinical symptoms [14–17]. The mechanisms underlying the regulation of ferroptosis have involved multiple aspects, exemplified by autophagy, iron metabolism, and reactive oxygen species (ROS) metabolism et al. [18–20]. Remarkably, system Xc^-^ as the unique antioxidant has attracted extensive concern of scholars.

Solute carrier family 7 member 11 (SLC7A11) is one of the subunits of the system Xc^-^, showing key modulation of iron overload-ferroptosis by transporting extracellular cysteine [12, 21]. Suppressing SLC7A11 reduced cystine uptake, which led to the deactivation of cystine-dependent glutathione peroxidase, enhanced intracellular lipid peroxidation and ferroptosis [22, 23]. Recently, it was reported that SLC7A11 is one of the critical targets of P53, and P53 could promote ferroptosis by inhibiting the uptake of cysteine [24–26]. Studies further confirmed that acetylation modulation of P53 is needed for the inhibition, and an acetylation defective mutant P53 with the specific mutated lysines to arginine residues could restore the SLC7A11 expression and improve the ferroptosis [27]. Therefore, targeting P53/SLC7A11 signaling may be a considered therapeutic approach to reverse ferroptosis during ALI.

Signal transducer and activators of transduction 6 (STAT6) is a key regulator in innate immune response, which mediates direct repression of inflammatory enhancers and regulates activation of alternatively polarization [28]. Series studies have showed that regulation of STAT6 could suppress inflammatory response by promoting M2 macrophages polarization [29–31]. Although the role of STAT6 in the field of immuno-regulation has been well studied, the function in intrinsic cells like lung epithelium remains unclear. In this study, we identified the indispensable role of STAT6 in maintaining alveolar epithelial cells homeostasis during ALI, moreover we originally investigated its novel regulatory mechanism of ferroptosis.

## Materials and methods

### Chemicals, antibodies and cell culture

Crystalline Silica particles (Quartz DQ 12) were purchased from Doerentrup Quarz GmbH (Germany). Lipopolysaccharide (LPS, SI732) was purchased from Beyotime (Shanghai, China). Ferrostatin-1 (Ferr-1, T6500) was purchased from TargetMol (MA, USA). Deferoxamine (DFO, GC13554), Erastin (GC16630), and RSL3 (GC12431) were purchased from Glpbio (Shanghai, China). Nicotinamide (NAM, 72340) was from Sigma Aldrich. TrichostatinA (TSA, S1045) was purchased from Selleck (Shanghai, China). Primary antibodies against STAT6 (sc-374021), p-STAT6 (sc-136019), PTGS-2 (sc-52972), P53 (sc-126), P21 (sc-6246), CBP (sc-32244), HA (sc-7392), Histone H3 (sc-517576), and β-actin (sc-47778) were from Santa Cruz (Texas, USA), and against SLC7A11 (AF7992) was from Beyotime (Shanghai, China). Antibody against 8-oxo-dG (#3154-MC-050) was from Trevigen (Gaithersburg, MD). Primary antibodies against Flag (#14793) and acetylated-lysine (#9441S) were purchased from Cell Signaling Technology (Danvers, MA, USA). Antibody against 4-hydroxynonenal (4-HNE) was from Bioss (Beijing, China). HRP-conjugated secondary antibodies were purchased from Immunoway (Plano, TX, USA; anti-mouse:RS0001, anti-rabbit:RS0002). Alexa Fluor 488 anti-mouse (ab150113, Abcam, UK) and Alexa Fluor 594 anti-rabbit antibodies (ab15008, Abcam, UK) and DAPI (Solarbio, China, C0065) were used in immunofluorescence (IF) staining. Human THP-1 acute monocytic leukemia cells and immortalized human bronchial epithelial HBE cells were purchased from ATCC (Manassas, VA, United States). THP-1 cells were cultured in RPMI1640 containing 10% fetal bovine serum (FBS, Hyclone), 1% penicillin/streptomycin (Invitrogen) and differentiated by 5 ng/ml phorbol-12-myristate-13-acetate (PMA, Sigma), while HBE cells were cultured in Dulbecco modified eagle medium (DMEM) supplemented with 10% FBS and 1% penicillin/streptomycin. The cells were maintained at 37 °C in a humidified incubator containing 5% CO_2_.

### Animal experiments

C57BL/6, STAT6^flox/flox^ and Sftpc^Cre^ mice were purchased from Cyagen Biosciences (Guangzhou, China). STAT6^flox/flox^ mice were crossed with Sftpc^Cre^ mice to generate lung epithelium-specific STAT6 knockout (STAT6^cKO^) mice. All mice received standard laboratory diet and maintained in 12 h light/dark cycle, climate-controlled and pathogen-free rooms. Mice handling in this study followed the Guide for the Care and Use of Laboratory Animals and the study protocols were approved by Soochow University Institutional Animal Care and Use Committee. Six to eight weeks old gender-matched wildtype and knockout mice from the same litter were selected randomly to indicated groups based on genotypes. Two in vivo studies were performed as follows: a) Mice were randomly divided into eight groups (*n* = 6 per group): (i) Control group (Ctrl), (ii) Crystalline Silica group (CS), (iii) Crystalline Silica and Ferrostatin-1 group (CS + Ferr-1), (iv) Crystalline Silica and Deferoxamine group (CS + DFO), (v) Lipopolysaccharide group (LPS), (vi) Lipopolysaccharide and Ferrostatin-1 group (LPS + Ferr-1), (vii) Lipopolysaccharide and Deferoxamine group (LPS + DFO), (viii) X-ray groups (X-ray). For the models of CS and LPS exposure, mice were anesthetized and intratracheally instilled with CS suspensions (3 mg/50 μl) or LPS (1 mg/kg). For the models of CS + Ferr-1/DFO, mice were intraperitoneally injected with Ferr-1 (1.25 µmol/kg) or intranasal instilled with DFO (10 mg/kg) for 7 consecutive days after CS instillation. For the models of LPS + Ferr-1/DFO, mice were pretreated with Ferr-1 or DFO for 2 consecutive days and then intratracheally instilled with LPS. Mice were sacrificed 24 h after LPS instillation. For the X-ray exposure model, mice were exposed to ionizing radiation (IR) at 20 Gy, which was delivered at the dose rate of 2 Gy/min and a source skin distance of 51 cm by an X-ray generator (Model X-RAD320iX; Precision X-Ray, Inc., North Branford, CT, USA), and sacrificed 3 days after radiation. All mice were euthanized and bronchoalveolar lavage fluid (BALF) was obtained by lavaging the whole lung with PBS (Invitrogen). b) STAT6 rescue in vivo study: Mice were intratracheally instilled with lenti-Veh or lenti-mouse STAT6 one week ago and at day 0 respectively. Mice from CS group were intratracheally instilled with CS at day 0 together with lentivirus administration. Model of LPS were intratracheally instilled with LPS 5 days after the second instillation of virus. All mice were sacrificed at day 7, and all biological samples were collected for following analyses.

### Hematoxylin and Eosin (H&E), Immunohistochemistry (IHC)

Lung tissues were fixed in 4% paraformaldehyde and the slides (4μm) were cut. H&E staining and lung injury scoring were performed as previously description [32–34]. For IHC staining, the lung sections were incubated with primary antibodies at 4 °C overnight. After washing three times with PBS, the slides were then stained with secondary antibody. The results were visualized by light macroscope (Leica DM 2500, Wetzlar, Germany).

### RNA extraction and quantitative real-time PCR (qRT-PCR)

Total RNA was extracted from lung tissues and cells using TRIzol reagent (CWBIO, Beijing, China). Equal amounts of RNA were reverse transcribed using HiFiScript cDNA synthesis kit according to the manufacturer’s instructions (CWBIO, Beijing, China) in 8-strip tubes (#404001, Nest, China). The real-time polymerase chain reaction was carried out using SYBR Green PCR Matser Mix (CWBIO, Beijing, China) according to the manufacturer’s protocol. The sequence of primers used in this study were listed in Supplementary Table 1.

### Immunoblot analysis, nuclear and cytoplasmic protein extraction, and Immunoprecipitation

For immunoblot analysis, lung tissues and cells were lysed with RIPA buffer and total protein content was quantified with bicinchoninic acid (BCA) protein assay kit (Fdbio science, Hangzhou, China). The extraction of nuclear and cytoplasmic protein was performed according to the manufacturer’s instructions (Fdbio science, Hangzhou, China). Briefly, lung tissues were homogenized and cells were lysed with reagent A in the kit. After vortex and incubation 20 min on ice, reagent B (1/20 volume of reagent A) was added. Followed by vortex and 1 min incubation on ice, the tissue or cell mixture were centrifuged 12,000 rpm, 4 °C for 15 min. Then the supernatant was harvested as cytoplasmic protein, and the precipitate was added with reagent N. After vortex and incubation on ice for 40 min, the precipitate was centrifuged and the supernatant was collected as nuclear protein. The lysates were denatured and electrophoresed through SDS-polyacrylamide gel and subjected to immunoblot analysis. For immunoprecipitation, cells were harvested in RIPA buffer (Fdbio science, Hangzhou, China) and pre-clear with 10 μl protein A agarose beads. After centrifugation, the supernatant was collected and added with indicated antibodies (1 μg) and rotation in 4 °C for 2 h. Then, the samples were added with another 20 μl protein A agarose beads and rotation in 4 °C overnight. Immunoprecipitated complexes were subjected to immunoblot with the indicated antibodies. Relative immunoblot bands were compared using the prestained protein marker (Vazyme Biotech Co.,Ltd, MP102-01) and Thermo Scientific PageRuler Prestained Protein Ladder (#26617).

### Transfection of shRNA and cDNA

Cells were transfected with vectors containing the indicated sh-RNA or with specific genes expression by PEI40000 reagent (40816ES03, Yeasen, China) according to the manufacturer’s instructions. Briefly, 1 μg of vector with 3 μl PEI40000 were separately mixed with 100 μl Opti-medium (Invitrogen) and combined with each other. After 20 min incubation, the mixtures were added into the cells. Cells were used for the following indicated studies after 24 or 48 h incubation (37 °C; 5% CO_2_).

### Response element (RE) of P53 on SLC7A11 promoter cloning

The sequence of human SLC7A11 promoter (−39 to −16) was synthetized in GENEWIZ and cloned into PGL3-Basic vector with KpnI and BgIII by a standard cloning protocol [35].

### Data collection

All data were downloaded from the National Center of Biotechnology Information (NCBI) Gene Expression Omnibus (GEO) (https://www.ncbi.nlm.nih.gov/geo/) [36]. The microarray-based expression data of lung tissue from rats with one week crystalline silica treatment were collected from GSE32147. Lung samples from wild type (WT) and Stat6^−/−^ mice were obtained from GSE1438. Raw count data of normal human lung tissue samples were obtained from the Genome Tissue Expression (GTEx) database [37].

### Bioinformatics analysis

Limma package was used to identify and filter differentially expressed genes (DEGs) between different groups in each of the datasets [38, 39]. In addition, R packages ggplot2 and Complex Heatmap were employed to visualize the DEGs [40]. The Gene sets and Gene Set Enrichment Analysis (GSEA) was performed by GSEA 4.1.0 to find possible functions of STAT6. Gene set enrichment analysis (GSEA) was carried out to verify the ferroptosis-related functions and explore the potential signaling pathway leading to ferroptosis between mice with high and low STAT6 expression [41, 42]. Protein-protein interactions (PPI) was performed by Retrieval of Interacting Genes/Proteins (STRING) to explain the potential regulation of STAT6 on ferroptosis [43]. The regulatory relationship between genes was visualized by Cytoscape 3.8.2 [44].

### Indirect immunofluorescence

Cells were seeded on glass coverslips (Thermo Fisher Scientific, Waltham, MA, USA). Cells were fixed with chilled methanol for 15 min, then coverslips were incubated by the primary antibodies and the respective secondary antibodies for 50 min each. Finally, the coverslips were mounted with antifade mounting solution (Invitrogen, Carlsbad, CA, USA), and images were acquired and observed with a fluorescence microscope (Leica DM 2500, Wetzlar, Germany).

### Detection of GSH, MDA and iron content

The content of GSH was detected by the corresponding commercial kit (A006-2-1, Nanjing Jiancheng Biotechnology, China) and MDA were measured using the Lipid Peroxidation MDA Assay Kit (S0131, Beyotime, China). The content of iron was measured by Iron Colorimetric Assay Kit (E-BC-K139-M, Elabscience, China). All kits were used under the manufacturer’s instructions.

### Cell viability assay

Cell viability was measured by MTT assay. 3-(4, 5-dimethylthiazol-2-yl)-2, 5-diphenyltetrazolium bromide was purchased from Sigma-Aldrich. Briefly, approximately 1 × 10^4^ cells per well were seeded in a 96-well plate for 48 h with multiply treatments. 20 μl MTT (2 mg/ml) in PBS solution was added and incubated for 3 h at 37 °C. The supernatant was removed and 100 μl of isopropanol/HCl was added. The absorbance at 570 nm was measured with a multifunctional microplate reader (Molecular Devices, USA).

### Dual-luciferase reporter assay

Cells were seeded in 24-wells plate and transfected with SLC7A11 promoter and other target plasmids using PEI40000 reagent (40816ES03, Yeasen, China). Luciferase activity was then evaluated with Dual Luciferase Reporter Gene Assay Kit (RG027, Beyotime, China) following the manufacturer’s instructions.

### Chromatin immunoprecipitation (ChIP)

HBE cells were fixed with formaldehyde, collected in PBS and re-suspended in SDS buffer. Cells were subsequently sonicated on ice. Following sonication, the lysates were then pre-cleared at 4 °C with protein A agarose, and then incubated with indicated antibodies and protein A agarose overnight. Antibody bead complexes were washed and eluted with corresponding buffer. Then cross-links were reversed and DNA was recovered. Then amounts of DNA in the complex were quantified by real-time PCR. Fold enrichment was calculated as ChIP signals divided by no antibody control and normalized to input. The primer sequence of human SLC7A11 used for ChIP was listed below:

Forward: AGGCTTCTCATGTGGCTGAT,

Reverse: AATAGCCACCAGCCTCTTCT

### Terminal deoxynucleotidyl transferase mediated dUTP nick end-labeling (TUNEL) assay

Lung sections or HBE cells were washed and fixed with 4% paraformaldehyde at room temperature. After incubation with proteinase K, lung sections or cells were subjected to TUNEL detection kit (Vazyme Biotech Co.,Ltd, A111). Samples were visualized by a fluorescence microscope (Leica DM 2500, Wetzlar, Germany).

### Lactate dehydrogenase (LDH) assay

LDH assay is usually used to measure the tissue or cell injury [45]. The LDH of mice BALF or of HBE cells was measured by the LDH assay kit (A020-2, Nanjing jiancheng Biotechnology, China) according to manufacturers’ instructions. Briefly, an aliquot of the BALF and HBE cell lysis were used to incubated with the reagents supplied by the above kit. After incubation for 5 min, absorbance was measured at 450 nm.

### Statistics

The investigators were blinded to group allocation. All related data were presented as means ± SD of three independent experiments performed in triplicate. GraphPad Prism 8.0 and R statistical software were performed for graphics and statistical analyses. For comparison between two groups, unpaired Student’s *t* tests were applied. Multiple comparisons were analyzed using one-way ANOVA with *Bonferroni’s* correction, and variance was similar between groups. *p* < 0.05 was considered statistically significant.

## Results

### STAT6 expression and activation are upregulated along with ferroptosis induction during ALI

To identify the role of ferroptosis in ALI, CS- and LPS- induced ALI mice models were established with Ferr-1 and DFO intervention. As shown in Supplementary Figs. S1A–D and S2A–D, PTGS-2 expression and iron content were both induced after CS and LPS exposure, which were inhibited by Ferr-1 and DFO administration. H&E staining showed obvious inflammatory cell infiltration and thickened alveolar septum in the lung tissues of CS- and LPS-treated mice, which were attenuated by Ferr-1 and DFO (Supplementary Figs. S1E,F and S2E,F). Next oxidative stress, as a critical component in the process of ALI was detected as well. As shown in Supplementary Figs. S1G–J and S2G–J, mice lung tissues were detected decreased GSH content but increased MDA content after CS and LPS exposure. Meanwhile, CS- and LPS- instillation also caused oxidative DNA damage as reflected by the upregulated 8-oxo-dG level (Supplementary Fig. S1K,L). Consistently, mice instilled with CS and LPS were also detected increased levels of protein and LDH in BALF (Supplementary Fig. S2K–N). Fortunately, all these damages above could be alleviated by Ferr-1 or DFO intervention (Supplementary Figs. S1 and S2). Additionally, TUNEL assay were performed to confirm whether other forms of cell death occurred in the ALI. The result of TUNEL staining showed that only few positive stained cells were observed after treated with CS or LPS (Supplementary Fig. S4). These data indicates that ferroptosis would be the major contributor to stimuli-induced ALI.

**Fig. 1:**
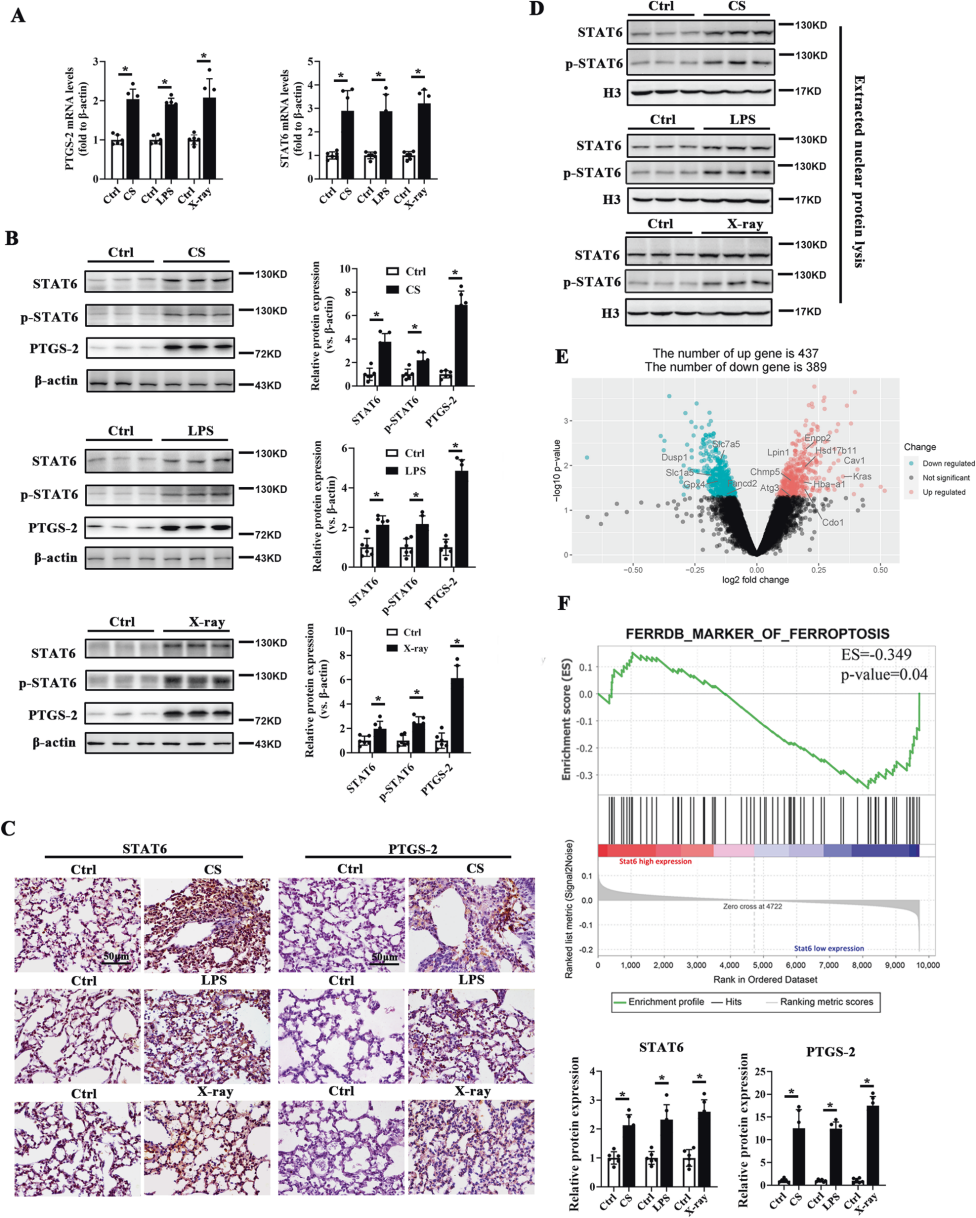
STAT6 is activated in CS, LPS and X-ray induced ferroptosis of acute lung injury. **A** The mRNA levels of PTGS-2 and STAT6 in indicated mice lung tissue were determined by qRT-PCR. **B** Lung tissue lysates from indicated group of mice were subjected to immunoblot analysis with the quantification on the right panel. **C** Representative photographs showed STAT6 and PTGS-2 staining in lung tissue from indicated groups. **D** The expression of STAT6 in indicated extracted nuclear protein. **E** Volcano plot showed differential expressed genes in rats with high and low Stat6 expression with ferroptosis-related genes labeled. **F** GSEA plot showed enrichment of “marker of ferroptosis” in STAT6 low expression group of rat. Data were expressed as means ± SD (*n* = 6, *p < 0.05, Ctrl *vs*. treatment).

Along with the induction of ferroptosis, STAT6 mRNA expression was upregulated with the stimuli (Fig. 1A). STAT6 protein level and activation were detected as well. The result showed that the protein levels of STAT6 and p-STAT6 were both increased after the stimuli exposure (Fig. 1B). Meanwhile, IHC staining showed that CS, LPS and X-ray exposure promoted the STAT6 translocation into the nuclear (Fig. 1C, Supplementary Fig. S3). Additionally, the extracted nuclear protein lysis was subjected to immunoblot and the result indicated that STAT6 was activated, manifesting as its increased expression in the nucleus (Fig. 1D). These data above indicates that STAT6 signals are increased along with the induction of ferroptosis during the above ALI.

### Ablation of STAT6 in pulmonary epithelium exacerbates ferroptosis and aggravates lung injury

To investigate the relationship between STAT6 and ferroptosis, we grouped CS-induced lung injury animal models (GSE32147) according to the median STAT6 expression and compared their gene expression profiles. A total of 826 genes (437 upregulated and 389 downregulated) was found to be significantly changed, which were labeled in the volcano map (Fig. 1E). The association between STAT6 and ferroptosis in lung tissue was further investigated through GSEA analysis. Marker genes of ferroptosis were observed significantly enriched in lung tissues with low expression of STAT6 (Fig. 1F). These results indicate that STAT6 is negatively correlated with ferroptosis in ALI.

**Fig. 2:**
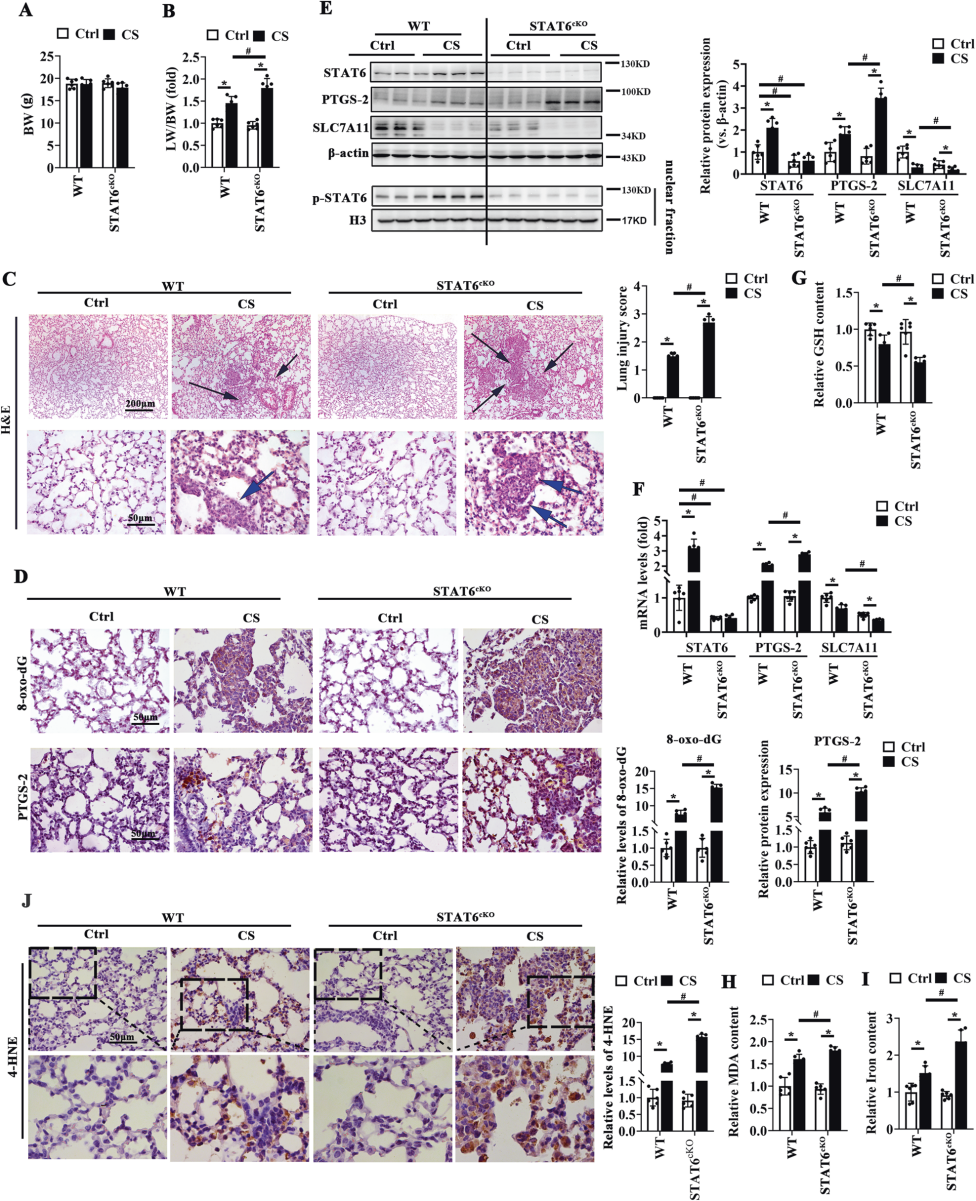
STAT6 deficiency in lung epithelium aggravates CS-induced ferroptosis and lung injury. Wild type (WT) and lung epithelium-specific STAT6 deficiency mice (STAT6^cKO^) mice were intratracheally instilled with CS and sacrificed 7 days later. **A** Body weight (BW) and **B** the ratio of lung weight to BW were determined. **C** Representative H&E-stained lung sections from WT and STAT6^cKO^ mice and lung injury score. (Black and blue arrows indicated inflammatory nodules and inflammatory cells infiltration respectively). **D** IHC staining of 8-oxo-dG and PTGS-2 of lung sections from indicated group were performed and quantified. Representative images from each group were shown. The protein expression (**E**) and mRNA **F** levels of STAT6, PTGS-2 and SLC7A11 in lung tissue were determined by western blot analysis and qRT-PCR assay. The relative GSH **G**, MDA (**H**) and Iron **I** content were measured by corresponding kit. **J** IHC staining of 4-HNE in lung sections from indicated group were performed and quantified. Representative images from each group were shown. The data were presented as means ± SD (*n* = 6, *p < 0.05, Ctrl *vs*. CS; ^#^*p* < 0.05, WT *vs*. STAT6^cKO^).

Next, lung epithelium-specific STAT6 deficient mice (STAT6^cKO^) generated by crossing STAT6^flox/flox^ and Sftpc^cre^ mice were also employed to build the ALI models. Tamoxifen was injected to induce the activity of cre accordingly [46]. The ratio of lung weight to body weight (LW/BW) were increased after CS instillation, and STAT6^cKO^ mice exhibited even higher LW/BW than WT mice (Fig. 2A,B). H&E staining showed that CS instillation induced the inflammatory cells infiltration and cellular nodules in the lung tissue of WT and STAT6^cKO^ mice, and STAT6 deficiency aggravated the inflammation (Fig. 2C). IHC staining of 8-oxo-dG showed that CS exposure caused oxidative DNA damage, which was exacerbated in STAT6^cKO^ mice (Fig. 2D). Meanwhile, STAT6^cKO^ mice were found to exhibit higher expression of PTGS-2 as identified by IHC staining, immunoblot and qRT-PCR analysis after CS instillation (Fig. 2D–F). Consistently, the downregulated GSH content caused by CS was further decreased in STAT6^cKO^ mice, and the increased MDA, iron content and 4-HNE were further upregulated (Fig. 2G–J). All these data indicates that CS-induced ferroptosis are aggravated in STAT6^cKO^ mice.

To verified whether STAT6 deficiency exacerbated ferroptosis is widely existed in ALI, LPS- and X-ray- induced ALI models were built as well. H&E staining showed that STAT6^cKO^ mice exhibited more inflammatory cell infiltration and thickened alveolar septum (Supplementary Figs. S5A and S6A) compared with WT mice after stimuli. Similarly, the induced levels of 8-oxo-dG and PTGS-2 by stimuli were further increased in STAT6^cKO^ mice lung tissues (Supplementary Figs. S5B, F and S6B, F). The suppressed content of GSH was even more decreased and the increased levels of MDA and iron accumulation were higher in STAT6^cKO^ mice lung tissues (Supplementary Figs. S5C–E and S6C–E). These data indicates that STAT6 deficiency in the lung epithelium promotes ferroptosis and exacerbates lung injury.

### STAT6 positively regulates SLC7A11 and suppresses ferroptosis

**Fig. 3:**
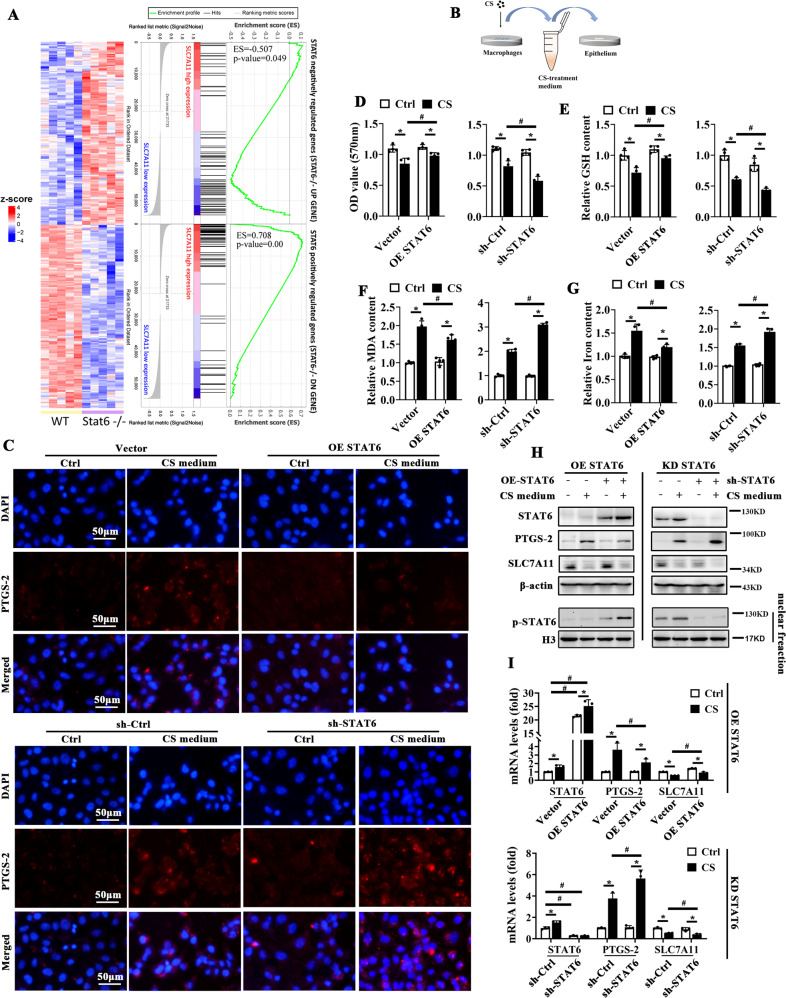
STAT6 suppresses ferroptosis and positively regulates SLC7A11. **A** Left panel, heat map showed genes negatively or positively regulated by STAT6. Right panel, GSEA plot showed enrichment of “STAT6 negatively regulated genes” in SLC7A11 low expression group and “STAT6 positively regulated genes” in SLC7A11 high expression group. **B** The co-cultured system of macrophages and epithelium. HBE cells were transfected with sh-Ctrl or plasmid for STAT6 inhibition or overexpression and treated with CS-medium for 24 h. **C** Immunofluorescence staining of PTGS-2 in cells with indicated treatment. DAPI was used for nucleus staining. The cell viability **D**, GSH **E**, MDA (**F**) and Iron **G** content in cells were measured (*n* = 4). The protein (**H**) and mRNA (**I**) levels of STAT6, PTGS-2 and SLC7A11 were measured by immunoblot analyses and qRT-PCR assay (*n* = 3). The data were presented as means ± SD (*p < 0.05, Ctrl *vs*. CS; ^#^*p* < 0.05, Vector/sh-Ctrl *vs*. OE-STAT6/sh-STAT6).

SLC7A11 is an critical ferroptosis related gene, and its expression was observed decreased in STAT6^cKO^ mice with or without stimuli (Fig. 2E,F, Supplementary Figs. S5F and S6F). In order to identify the relationship between STAT6 and SLC7A11, the gene sets of STAT6 knockout mice were employed to apply GSEA to GTEx samples grouped by median SLC7A11 expression. In GSE1438, the 150 significant DEGs with the maximum log2FC were defined as genes negatively regulated by STAT6. Simultaneously, the 150 significant DEGs with the minimum log2FC were defined as genes positively regulated by STAT6. The expression of 300 DEGs selected into gene sets based on the expression profile of GSE1438 was shown in the heat map (Fig. 3A left). Among them, there were 77 upregulated genes and 98 downregulated genes finally mapped to homologous genes of homo sapiens. Then, GSEA revealed that genes negatively regulated by STAT6 were significantly enriched in SLC7A11 low expression group. Correspondingly, genes positively regulated by STAT6 were enriched in SLC7A11 high expression group (Fig. 3A right). These results suggest that SLC7A11 is positively correlated with STAT6 signaling.

To investigate the detail regulation of STAT6 on ferroptosis, the differentiated THP-1 cells were exposed with CS for 24 h and the cell culture medium was harvested to further treat HBE cells (Fig. 3B). CS medium treatment upregulated the expression of PTGS-2, which was further increased by silencing STAT6 but decreased by STAT6 overexpression (Fig. 3C, H, I). Consistently, STAT6 overexpression restored the cell viability and GSH content, while silencing STAT6 reversed these changes (Fig. 3D, E). Also, CS medium incubation dramatically increased the MDA and iron content, which was alleviated with STAT6 overexpression but exacerbated with STAT6 knockdown (Fig. 3F,G). The results above were consistently observed in the cells treated with LPS (Supplementary Fig. S7A–F). Additionally, the classical ferroptois inducer Erastin and RSL3 were also used to confirmed the role of STAT6 in regulating ferroptosis. It was shown that the induced content of iron and MDA was further increased and the decreased GSH level was further suppressed after inhibiting STAT6 (Supplementary Fig. S7G–I). Besides, SLC7A11 expression was decreased after CS medium or LPS treatment, which was restored with STAT6 overexpression but aggravated with STAT6 knockdown (Fig. 3H–I, Supplementary Fig. S7F). These results indicate that STAT6 restores the depressed SLC7A11 expression in ferroptosis and plays an important role in suppressing ferroptosis.

### STAT6 downregulates P53 signaling to alleviate ferroptosis through decreasing its acetylation modification

**Fig. 4:**
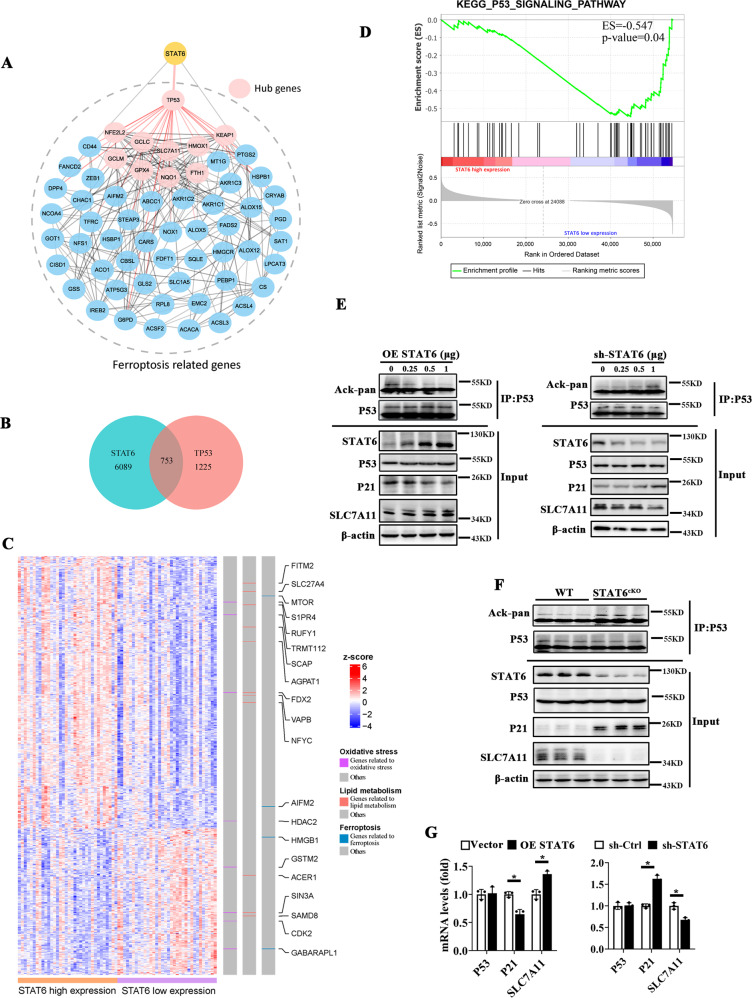
STAT6 downregulates P53 signaling pathway to alleviate the ferroptosis by decreasing its acetylation modification. **A** PPI network showed the relationship between STAT6 and ferroptosis related genes. Red nodes represented hub genes calculated by plugin Cytohubba. Red edges represented interactions between TP53 and its associated genes. **B**–**D** were performed upon GTEx lung tissue sample. **B** Venn diagram showed the overlap between 6089 genes that were significantly differentially expressed grouped by STAT6 and 1225 genes grouped by TP53. **C** Heat map showed the expression of 753 common DEGs in GTEx samples with genes associated with specific biological process annotated. **D** GSEA plot showed enrichment of KEGG term “P53 signaling pathway” in STAT6 low expression group. **E** HBE cells were transfected with indicated plasmid and harvested for immunoprecipitation and immunoblot analysis with the indicated antibodies. **F** The protein lysis of lung tissue from WT and STAT6^cKO^ mice were subjected to immunoprecipitation and immunoblot analysis with the indicated antibodies. **G** The mRNA levels of P53, P21, SLC7A11 were measured by qRT-PCR assay. (*n* = 4, *p < 0.05, Vector/sh-Ctrl *vs*. OE-STAT6/sh-STAT6).

To further explore the possible regulation mechanism of STAT6 on ferroptosis, we selected STAT6 and ferroptosis related proteins reported in literature to establish protein-protein interaction (PPI) network and calculate the top 10 genes as the hub genes indicated [47]. The network diagram showed that P53 is the only hub gene associated with STAT6 and was closely related to other nodes in the network, which indicated that STAT6 may regulate other ferroptosis through P53 (Fig. 4A). Next, we performed differential analysis separately on GTEx lung tissue data categorized by median STAT6 and P53 expression. The results demonstrated that there were 753 overlaps between significant differentially expressed grouped by STAT6 and P53 (*p* < 0.05) (Fig. 4B). Then, according to STAT6 expression level, the expression condition of 753 common DEGs in 68 GTEx samples was revealed in Fig. 4C, among which the genes related to oxidative stress, lipid metabolism and ferroptosis were annotated on the side strip. Additionally, we also found that P53 signaling pathway geneset from Kyoto Encyclopedia of Genes and Genomes (KEGG) database was significantly enriched in the group with low STAT6 expression by GSEA (Fig. 4D). These findings suggest that STAT6 attenuates ferroptosis in lung injury by negatively regulating P53 signaling.

Subsequently, the potential regulation of STAT6 on P53 signaling and its association with SLC7A11 were detected. HBE cells were transfected with STAT6 and P53 plasmid, then the cells were harvested and subjected to immunoprecipitation and immunoblot analysis. The results showed that there was no direct binding between STAT6 and P53, meanwhile the location of P53 was not affected by STAT6 (Supplementary Fig. S8A–C). Next the results of immunoprecipitation (Fig. 4E) and qRT-PCR (Fig. 4G) showed that the protein and mRNA levels of P21 as well as acetylated P53 but not P53 itself were dose-dependently decreased with STAT6 overexpression, which were increased by STAT6 inhibition oppositely. The expression of SLC7A11 was positively regulated by STAT6 in the cells. Besides, in order to confirmed that STAT6 specifically regulated the acetylation of P53, we additionally conducted the IP assay using the Ack-pan antibody to detect P53 expression, and the results consistently showed that STAT6 negatively regulated P53 acetylation (Supplementary Fig. S8D). Similarly, the protein levels of P53 acetylation and P21 were significantly increased in STAT6^cKO^ mice lung tissues, while the protein expression of SLC7A11 was decreased compared to WT mice (Fig. 4F). Furthermore, we confirmed whether P53 acetylation regulated CS or LPS induced iron accumulation and lipid peroxidation. As shown in Supplementary Fig. S8F,G, induction of P53 acetylation using TSA/NAM [48] upregulated CS and LPS induced iron and MDA content, while suppressing P53 acetylation by silencing CBP downregulated the increased iron and MDA content. Taken together, the results suggest that STAT6 alleviates ferroptosis may be through inhibiting P53 acetylation to improve the expression of SLC7A11.

### STAT6 competitively binds with CBP and restores the inhibition of P53 on SLC7A11 expression

**Fig. 5:**
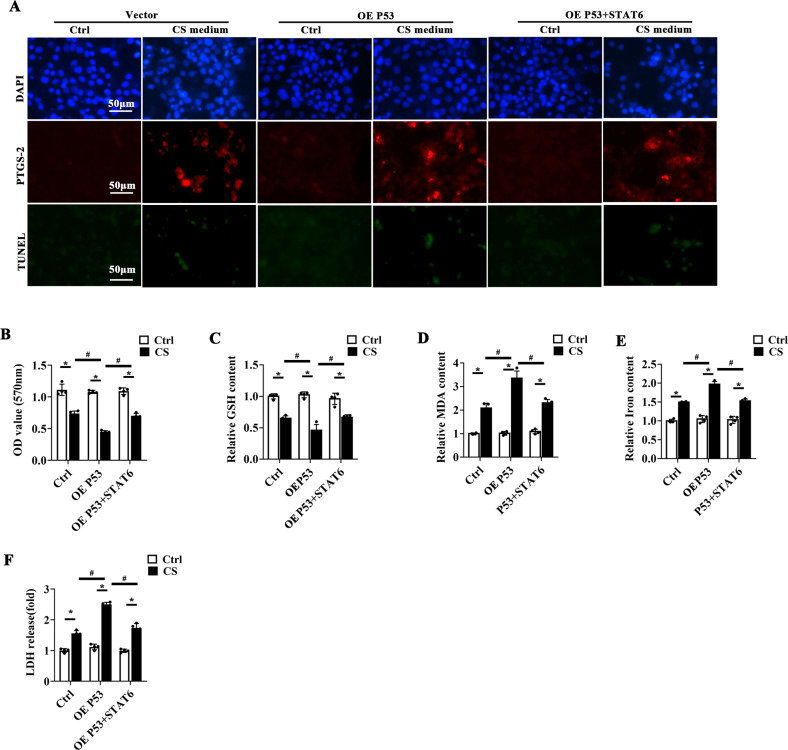
STAT6 combats P53 overexpression induced cell ferroptosis. HBE cells were transfected with empty vector or plasmids for P53 and/or STAT6 overexpression. Then cells were treated with Ctrl or CS medium for another 24 h. **A** Representative images of PTGS-2 immunofluorescence and TUNEL staining of cells from the indicated group. The cell viability **B**, GSH **C**, MDA **D**, Iron **E** content and LDH release (**F**) of indicated group were measured by corresponding kit. The data were presented as means ± SD (*n* = 4, *p < 0.05, Ctrl *vs*. CS; ^#^*p* < 0.05, P53 + CS *vs*. CS or P53 + STAT6 + CS).

**Fig. 6:**
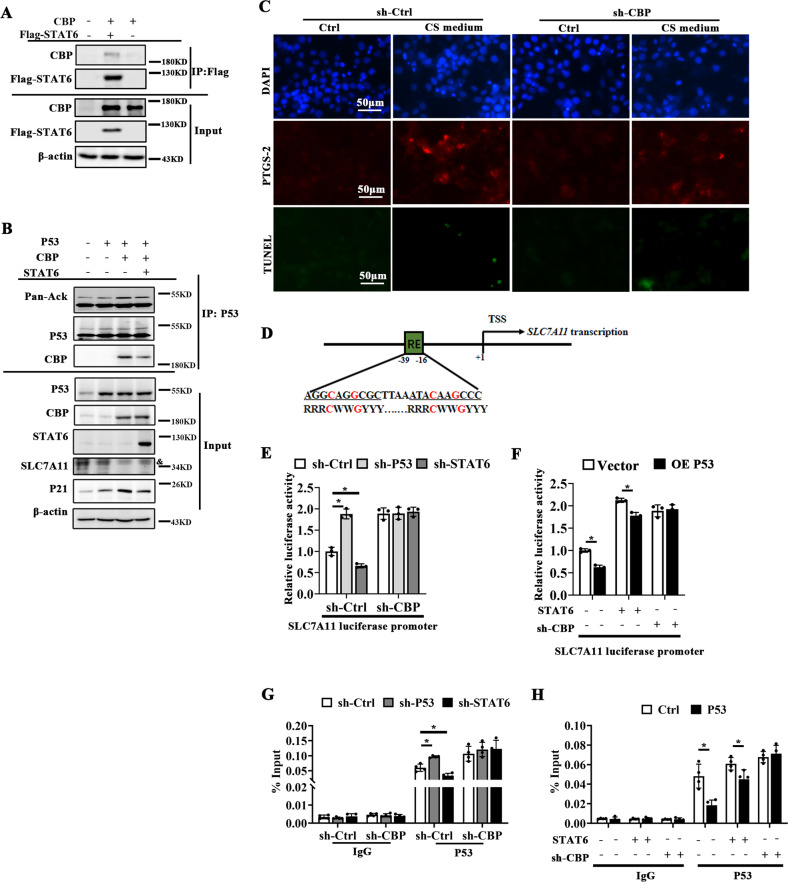
STAT6 competitively binds with CBP to restore the inhibition of P53 on the expression of SLC7A11. **A** Cells transfected with difference combination of CBP and Flag-STAT6 were subjected to immunoprecipitation (IP) followed by immunoblot analyses. **B** HBE cells were co-transfected with different combination of P53, CBP, STAT6 as indicated and subjected to immunoprecipitation (IP) followed by immunoblotting (& means the indicated band). **C** Cells transfected with sh-Ctrl or sh-CBP were subjected to immunofluorescence staining of PTGS-2 and TUNEL assay. Representative images were shown. **D** Schematic diagram of P53 binding site and sequence on human SLC7A11 gene. (R, A/G; W, A/T; Y, C/T; nucleotides C and G in red are essential for P53 binding). TSS, transcription start site. **E** HBE cells were transfected with difference combination of sh-CBP, sh-P53, sh-STAT6 accordingly and the luciferase activity of SLC7A11 was detected. Data were presented as means ± SD (*n* = 3, *p < 0.05, Ctrl vs. treatments). **F** The effects of P53 overexpression on the context of STAT6 overexpression or sh-CBP. Luciferase activity of SLC7A11 was determined. The results were presented as means ± SD (*n* = 3, *p < 0.05, Vector *vs*. OE-P53). **G**,**H** The regulation of P53 on SLC7A11 promoter was confirmed by ChIP assay in HBE cells with indicated treatment. Results were expressed as mean ± SD (*n* = 4 *p < 0.05, sh-Ctrl *vs*. sh-P53/sh-STAT6 or Ctrl *vs*. P53 overexpression).

To confirm that STAT6 regulates ferroptosis via P53, HBE cells transfected with P53 and STAT6 were subjected to analysis. In accordance with the results of Fig. 4, P53 overexpression aggravated CS-induced ferroptosis as determined by the increased level of PTGS-2 and decreased cell viability, which could be inversely improved by STAT6 overexpression (Fig. 5A,B). Consistently, CS-induced GSH inhibition (Fig. 5C), MDA generation (Fig. 5D), iron accumulation (Fig. 5E) and LDH release (Fig. 5F) were all exacerbated with P53 overexpression, which were alleviated by STAT6 co-transfection. Next, the molecular mechanism of STAT6-induced ferroptosis resistance were further explored. P53 acetylation is the critical modulation related to its interaction with response elements (RE) to regulate its targets transcription, and CBP is the key acetyltransferase for P53 acetylation [49]. The interaction between CBP and STAT6 was first identified (Fig. 6A). Silencing CBP suppressed CS-induced PTGS-2 expression but increased the luciferase activity of SLC7A11 promoter (Fig. 6C–E). Moreover, immunoprecipitation assay suggested that STAT6 overexpression competitively bound with CBP, decreasing the binding between P53 and CBP, inhibiting P53 acetylation (Fig. 6B). Additionally luciferase reporter assay showed that the inhibited activity of SLC7A11 promoter by P53 were attenuated by STAT6 overexpression or CBP knockdown, which was confirmed by ChIP assay (Fig. 6F–H). Besides, the IP analysis also indicated that knockdown of CBP changed p53 acetylation and SLC7A11 expression (Supplementary Fig. S8E). These data indicates that STAT6 competitively binds with CBP to restore the inhibition of P53 on SLC7A11 expression to improve ferroptosis.

### Rescue of STAT6 inhibits ferroptosis and attenuates lung injury in both CS- and LPS-induced models

**Fig. 7:**
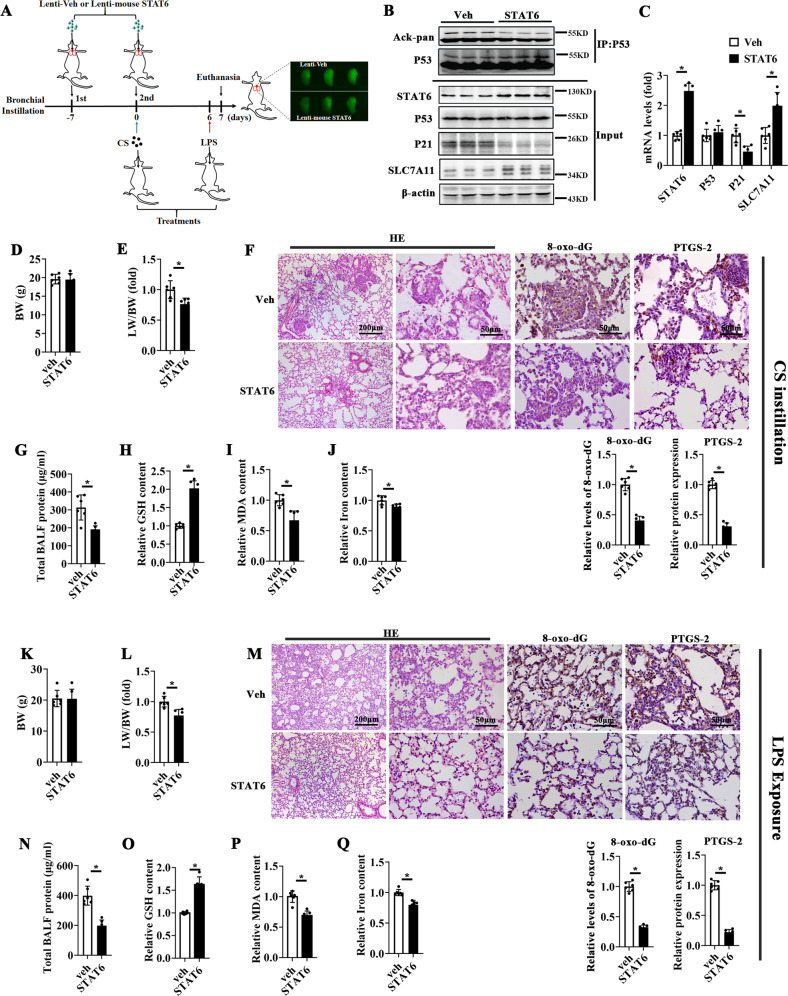
Rescue of STAT6 attenuates ferroptosis and acute lung injury in both CS- and LPS-induced models. **A** Timeline of lentivirus transduction with indicated treatment. Mice were intratracheally instilled with lenti-Veh or lenti-mouse STAT6 twice at one week ago and at day 0 respectively. Then Veh and STAT6 mice were bronchially instilled with CS at day 0 or LPS at day 6. All mice were sacrificed at day 7. Lentivius labeled with GFP was successfully instilled as confirmed by the photographs right panel. **B** Lung tissue lysates were harvested and subjected to immunoprecipitation (IP) followed by immunoblot analysis. Representative binds were shown. **C** The mRNA levels of STAT6, P53, P21, SLC7A11 from Veh or STAT6 mice were determined by qRT-PCR assay (*n* = 6). (D-H) Mice were co-treated with lenti-Veh or lenti-mouse STAT6 along with or without CS instillation. Body Weight (**D**) and LW/BW **E** were determined. **F** Representative H&E-stained images and IHC staining of 8-oxo-dG and PTGS-2 of lung sections. Total BALF protein (**G**) and relative GSH **H**, MDA (**I**) and Iron **J** content in lung tissue were measured by corresponding kit. Mice were co-treated with lenti-Veh or lenti-mouse STAT6 along with or without LPS. BW (**K**) and LW/BW **L**, H&E, IHC staining of 8-oxo-dG and PTGS-2 of lung sections **M**, total BALF protein (**N**), and relative GSH **O**, MDA (**P**) and Iron **Q** content in lung tissues. The results were presented as means ± SD (*n* = 6, *p < 0.05, Ctrl *vs*. treatments).

Next, lentivirus-mediated overexpression of STAT6 were evaluated in CS- and LPS-induced ALI models. The fluorescence images of lung tissues indicated the infection efficiency in both lenti-Veh and lenti-mouse STAT6 groups (Fig. 7A). The expression of STAT6 in the lung tissue of lenti-mouse STAT6 instilled mice was dramatically induced as showed in Fig. 7B, C. And lenti-mouse STAT6 treatment reduced LW/BW (Fig. 7E, L) and BALF protein (Fig. 7G, N). Besides, H&E and IHC staining of 8-oxo-dG showed that the pathological damage and oxidative stress were restored by STAT6 rescue (Fig. 7F, M). Furthermore, STAT6 rescue alleviated CS and LPS caused ferroptosis, manifesting as the decreased expression of PTGS-2 (Fig. 7F, M), the restored GSH content as well as the suppressed content of MDA and iron (Fig. 7H–J, O–Q). These findings indicate that rescue of STAT6 mitigates ferroptosis and improves ALI.

## Discussion

Ferroptosis is recently emerging as a new form of cell death, characterized by intracellular iron accumulation and lipid peroxidation [12]. Compelling evidences have indicated the role of ferroptosis in the pathophysiological process of acute injury in multiple tissues especially lung tissue [50–52]. Inhibition ferroptosis could decrease the tissue injury accordingly. Liu et al. reviewed that sevoflurane protects against LPS-induced acute lung injury by inhibiting ferroptosis [53]. Li et al. reported that panaxydol attenuates ferroptosis against LPS-induced acute lung injury in mice [54]. Although studies supported that ferroptosis offers new perspective for the treatment of ALI, whether it is widely existed in most of ALI and its detail regulatory mechanism remains unclear. In this study, Ferr-1 and DFO, as classical inhibitor of ferroptosis, were both used to confirm the significant contribution of ferroptosis to three ALI mice models caused by three common stimuli, including physical factors (CS and X-ray) and biological factor (LPS). All of the three models were detected obvious lung injury, along with increased content of iron and MDA, upregulated expression of PTGS-2 as well as decreased GSH, which were attenuated by Ferr-1 and DFO intervention (Supplementary Figs. S1 and S2). Although TUNEL assay showed that there might be some other forms of cell death during the ALI, ferroptosis still a major contributor as demonstrated by much more positive PTGS-2 staining cells after the stimuli. Thus, exploring the underlying regulation of ferroptosis is imperative for ALI therapeutic strategies development.

STAT6 is a type 2 regulator, and its role in immuno-regulation has been well established. However, its function in intrinsic cells like lung epithelial cells, the most susceptible and the first affected cells during ALI has not been well investigated. Different from the previous studies that STAT6 regulates macrophages clearance of apoptotic neutrophils and resolve LPS-induced ALI [55], here we focused on the regulation of STAT6 on ferroptosis of lung epithelial cells. We originally found that STAT6 expression and activation were upregulated with the increased ferroptosis in the above ALI (Fig. 1). Then the negative association of STAT6 and ferroptosis was further demonstrated through bioinformatic analysis (Figs. 3 and 4).

In order to clarify the impact of STAT6 on ferroptosis of ALI, we generated epithelium-specific STAT6 deficiency mice (STAT6^cKO^). Obviously, STAT6 ^cKO^ mice exhibited exaggerated ferroptosis and more serious lung injury when exposed to the stimuli (Fig. 2, Supplementary Fig. S5–6). Consistently, STAT6 knockdown in vitro also exhibited more serious damage, while STAT6 overexpression attenuated the ferroptosis (Fig. 3). These results indicated that STAT6 activation negatively regulates ferroptosis in ALI. On the basis of our current study and previous reports, we thereby proposed that the improvement of STAT6 on ALI may be mostly due to its suppression on epithelium ferroptosis.

The guardian P53, encoded by the TP53 gene, has been primarily linked to its canonical functions including induction of cell-cycle arrest, senescence, and apoptosis [56]. While recent studies have reported the non-canonical functions of P53 such as controlling metabolism and redox state, which are closely related with ferroptosis regulation [57, 58]. P53 has either pro- or anti-ferroptotic functions in response to oxidative stress [59]. Studies have shown that under basal or low ROS stress, P53 might serve as a rheostat by upregulating Nrf2 pathway, preventing ferroptosis. However, under high oxidative stress, the induction of P53 promotes ferroptosis, which always causes the tissue injury [60, 61]. In this study, we consistently found that overexpression P53 promotes HBE cells ferroptosis and deteriorates cell damage (Fig. 5).

Furthermore, it has been reported that P53 sensitizes cells to ferroptosis by transcriptional suppression of SLC7A11, inhibiting the cystine uptake and decreasing the GSH levels [25]. Consistently, we found that P53 was activated in the ALI to trigger ferroptosis in response to ROS stress (Supplementary Fig. S8). Remarkably, previous studies showed that the P53 acetylation is necessary for its regulation on targets. Wang et al. reported that regulation of SLC7A11 expression requires acetylation of the DNA-binding domain of P53 [27]. Unexpectedly, some studies showed that an acetylation defective mutant P53 with three mutated lysines (K117/161/162R) failed to induce apoptosis, senescence, and cell-cycle arrest, but still sensitized the cells to ferroptosis [25, 56]. Alternatively, ectopic expression of the quadruple acetylation defective mutant of P53 in P53-null cells showed that it failed to inhibit SLC7A11 and regulate ferroptosis accordingly [27]. Previous studies have also showed that spermidine/spermine N1-acetyltransferase and CBP are associated with P53 acetylation [62, 63]. Here, we demonstrated that silencing CBP increased SLC7A11 transcription (Fig. 6). Specifically, STAT6 competitively bound with CBP, which suppressed the association of P53 and CBP and decreased the acetylation of P53. Thus, STAT6 negatively regulated ferroptosis via regulating P53/SLC7A11 pathway. In corporation with the induction of P53 in the ALI, modulation intracellular STAT6 was found to attenuate the P53-mediated ferroptosis and increase the cell anti-oxidant capability (Fig. 5).

**Fig. 8:**
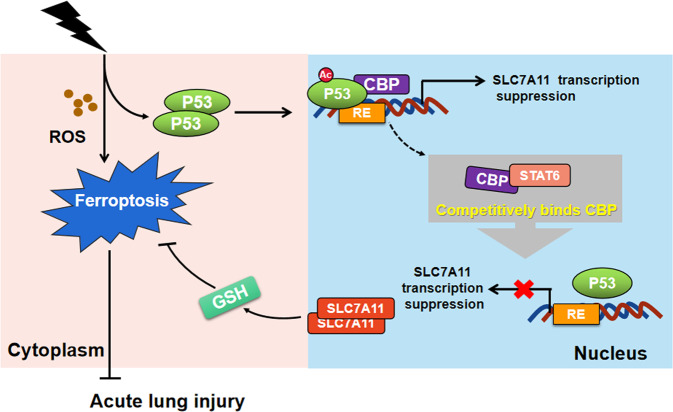
Proposed model for the regulation of STAT6 on ferroptosis. STAT6 competitively binds with CBP, suppressing the association of P53 and CBP, decreasing the acetylation of P53, restoring SLC7A11 expression and alleviating acute lung injury.

In summary, our present study originally revealed the competitive binding between STAT6 and CBP, which serves as a crucial event that decreases P53 acetylation, restoring its inhibition on SLC7A11, and finally inhibits ferroptosis (Fig. 8). Our results may provide a potential therapeutic for treating ALI.

## Supplementary information

Supplementary information

Figure S1

Figure S2

Figure S3

Figure S4

Figure S5

Figure S6

Figure S7

Figure S8

Original Data File

checklist

## Data Availability

The data that support the findings of this study are included in this published article and its supplementary information files are available from the authors on reasonable request, see author contributions for specific data sets.
