# Supplementary material for: STAT6 inhibits ferroptosis and alleviates acute lung injury via regulating P53/SLC7A11 pathway
Source: Cell Death Dis. 2022 Jun 6;13(6):530. doi: 10.1038/s41419-022-04971-x (PMC9169029; doi:10.1038/s41419-022-04971-x)
Supplement: Supplementary file 1 — Supplementary information [file 41419_2022_4971_MOESM1_ESM.docx]

**Supplementary information**

**Supplementary figure legends**

**Figure S1. Ferroptosis is identified in CS-, LPS-induced ALI using Ferr-1.** Mice were co-treated with CS or LPS along with Ferr-1. (A, B) IHC staining of PTGS-2 in lung tissue. (C, D) Relative iron content in indicated group. (E, F) Representative H&E-staining of lung sections from indicated group. (The black and blue arrows indicated inflammatory nodules and inflammatory cells infiltration respectively). Relative GSH (G, I), MDA (H, J) content of lung tissue from indicated group of mice. (K, L) IHC staining of 8-oxo-dG of lung sections and quantification were performed. The data were presented as means ± SD (n=6,**p* < 0.05, Ctrl *vs.* treatment; #*p* < 0.05, CS *vs.* CS+Ferr-1, LPS *vs.* LPS+Ferr-1).

**Figure S2. Ferroptosis is confirmed in CS-, LPS-induced ALI using DFO.** Mice were co-treated with CS or LPS along with DFO. (A, B) IHC staining of PTGS-2 in lung tissue. (C, D) Relative iron content in indicated group. (E, F) Representative H&E-staining of lung sections from indicated group. (Black arrows indicated inflammatory nodules and blue arrows indicated inflammatory cells infiltration). Relative GSH (G, I), MDA (H, J) content in indicated mice lung tissue. Total protein (K, M) and relative LDH content (L, N) in BALF. The data were presented as means ± SD (n=6,**p* < 0.05, Ctrl *vs.* treatment; #*p* < 0.05, CS *vs.* CS+DFO, LPS *vs.* LPS+DFO).

**Figure S3. STAT6 is activated along with the increased expression of PTGS-2.** Lung tissue sections from indicated group of mice were subjected to IHC staining. Representative photographs showed STAT6 and PTGS-2 staining in lung tissue from indicated group with the amplification on the right panel.

**Figure S4. The detection of TUNEL assay by CS or LPS treatments.** Mice were co-treated with CS or LPS and Ferr-1. Lung tissue sections from indicated group of mice were subjected to TUNEL assay and representative images were shown.

**Figure S5. STAT6 deletion aggravates LPS-induced ferroptosis.** WT and STAT6^cKO^ mice were received LPS intratracheally instillation. (A) Representative H&E-staining of lung sections from WT and STAT6^cKO^ mice. (The arrows indicate inflammatory cells infiltration). (B) IHC staining of 8-oxo-dG and PTGS-2 of lung sections from indicated group were performed and quantified. Representative images were shown. The relative GSH (C), MDA (D) and Iron content (E) in lung tissue were measured by corresponding kit. (F) Lung tissue lysates from indicated group of mice were subjected to immunoblot analysis and representative images were shown. The data were presented as means ± SD (n=6,**p* < 0.05, Ctrl *vs.* LPS; #*p* < 0.05, WT *vs.* STAT6^cKO^).

**Figure S6. STAT6 deficiency exacerbates ferroptosis in** **X-ray induced ALI.** WT and STAT6^cKO^ mice were exposed to X-ray. (A) Representative H&E-staining of lung sections from WT and STAT6^cKO^ mice. (The arrows indicate inflammatory cells infiltration). (B) IHC staining of 8-oxo-dG and PTGS-2 of lung sections from indicated group were performed and quantified. Representative images were shown. The relative GSH (C), MDA (D) and Iron content (E) in lung tissue were measured by corresponding kit. (F) Lung tissue lysates from indicated group of mice were subjected to immunoblot analysis with indicated antibodies and representative images were shown. The data were presented as means ± SD (n=6,**p* < 0.05, Ctrl *vs.* X-ray; #p < 0.05, WT *vs.* STAT6^cKO^).

**Figure S7. STAT6 attenuated LPS-induced ferroptosis with the improvement of SLC7A11 expression.** HBE cells were transfected with sh-Ctrl or plasmids for STAT6 inhibition or overexpression and followed with LPS 24h treatmeant. (A) Immunofluorescence staining of PTGS-2 in cells with indicated treatment. DAPI was used for nucleus staining. The cell viability (B), GSH (C), MDA (D) and Iron (E) content of indicated group were performed using corresponding kit. (F) The protein expression levels of STAT6, PTGS-2 and SLC7A11 were measured by immunoblot analyses. (G-I) HBE cells were transfected with sh-Ctrl or sh-STAT6 and then treated with Erastin or RSL3. Relative Iron, GSH and MDA content were measured using corresponding kit. The results were presented as means ± SD (n=4, **p* < 0.05, Ctrl *vs.* treatments; #*p* < 0.05, Vector/sh-Ctrl *vs.* OE STAT6/sh-STAT6).

**Figure S8. STAT6 has no direct binding with P53 but CBP, which affects the acetylation of P53.** (A) HBE cells were transfected with HA-STAT6 and P53 accordingly. Cell lysates were harvested and subjected to immunoprecipitation (IP) and immunoblot analyses. (B) The cytoplasmic and nuclear protein were separated from above treated cells and subjected to immunoblot with indicated antibodies. (C) Cells transfected with P53 and/or STAT6 were subjected to immunofluorescence staining of STAT6 and P53. And the ratio of P53 colocalized with DAPI was quantified. The results were presented as means ± SD (n=3). (D) HBE cells were transfected with indicated plasmids and harvested for immunoprecipitation and immunoblot analysis with the indicated antibodies. (E) Cells transfected with sh-Ctrl or sh-CBP were subjected to CS medium or LPS. Cell lysates were harvested for immunoprecipitation (IP) and immunoblot analyses. (F-G) Relative Iron and MDA content in cells with indicated treatment. The results were presented as means ± SD (n=4, **p* < 0.05, Ctrl *vs.* Treatments)

**Table 1. Primer sequence for qRT-PCR**

| Primer Sequence (5’-3’) | | |
| --- | --- | --- |
| m-STAT6 | Forward | CTCTGTGGGGCCTAATTTCCA |
|  | Reverse | CATCTGAACCGACCAGGAACT |
| m-PTGS-2 | Forward | AGTCTTTGGTCTGGTGCCTG |
|  | Reverse | GTTGCTCATCACCCCACTCA |
| m-SLC7A11 | Forward | GTCATCGGATCAGGCATCTT |
|  | Reverse | CATAGGACAGGGCTCCAAAA |
| m-P53 | Forward | TGGAAGACTCCAGTGGGAAC |
|  | Reverse | TCTTCTGTACGGCGGTCTCT |
| m-P21 | Forward | TTGCACTCTGGTGTCTGAGC |
|  | Reverse | AATCTGTCAGGCTGGTCTGC |
| m-β-actin | Forward | AAGGCCAACCGTGAAAAGAT |
|  | Reverse | GTGGTACGACCAGAGGCATAC |
| h-STAT6 | Forward | GTCTGGTCTCCAAGATGCCC |
|  | Reverse | ATATGCTCTCAAGGGTGCTGA |
| h-PTGS-2 | Forward | AGGGTTGCTGGTGGTAGGAA |
|  | Reverse | TTCATCTGCCTGCTCTGGTC |
| h-SLC7A11 | Forward | CCTTTCAAGGTGCCACTGTT |
|  | Reverse | AGTGATGACGAAGCCAATCC |
| h-P53 | Forward | AGGCCTTGGAACTCAAGGAT |
|  | Reverse | TGAGTCAGGCCCTTCTGTCT |
| h-P21 | Forward | GGAAGACCATGTGGACCTGT |
|  | Reverse | GGCGTTTGGAGTGGTAGAAA |
| h-β-actin | Forward | CTGGGACGACATGGAGAAAA |
|  | Reverse | AAGGAAGGCTGGAAGAGTGC |
