# Supplementary figures and images for: STAT6 inhibits ferroptosis and alleviates acute lung injury via regulating P53/SLC7A11 pathway
Source: Cell Death Dis. 2022 Jun 6;13(6):530. doi: 10.1038/s41419-022-04971-x (PMC9169029; doi:10.1038/s41419-022-04971-x)

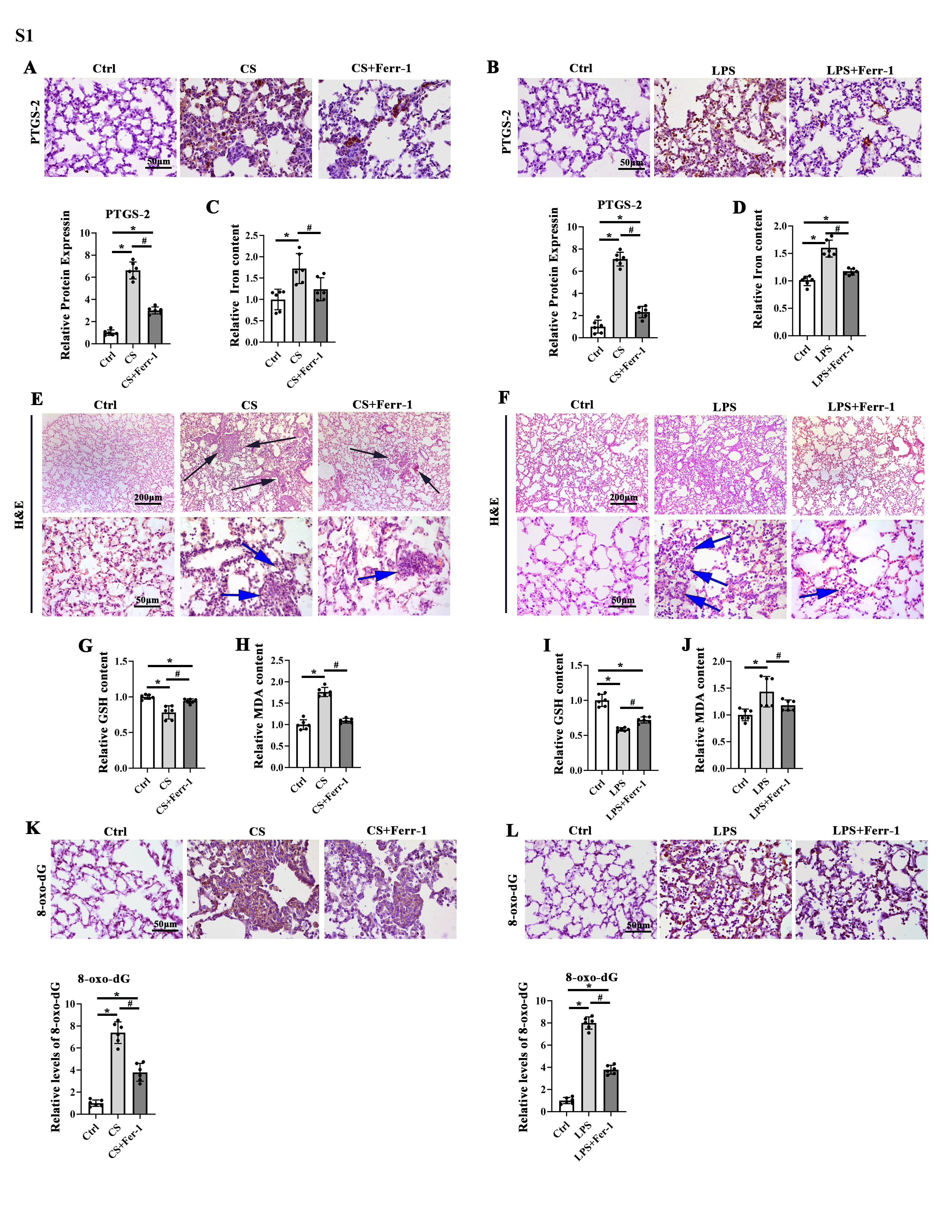

Supplement: Supplementary file 2 — Figure S1 [file 41419_2022_4971_MOESM2_ESM.tif]

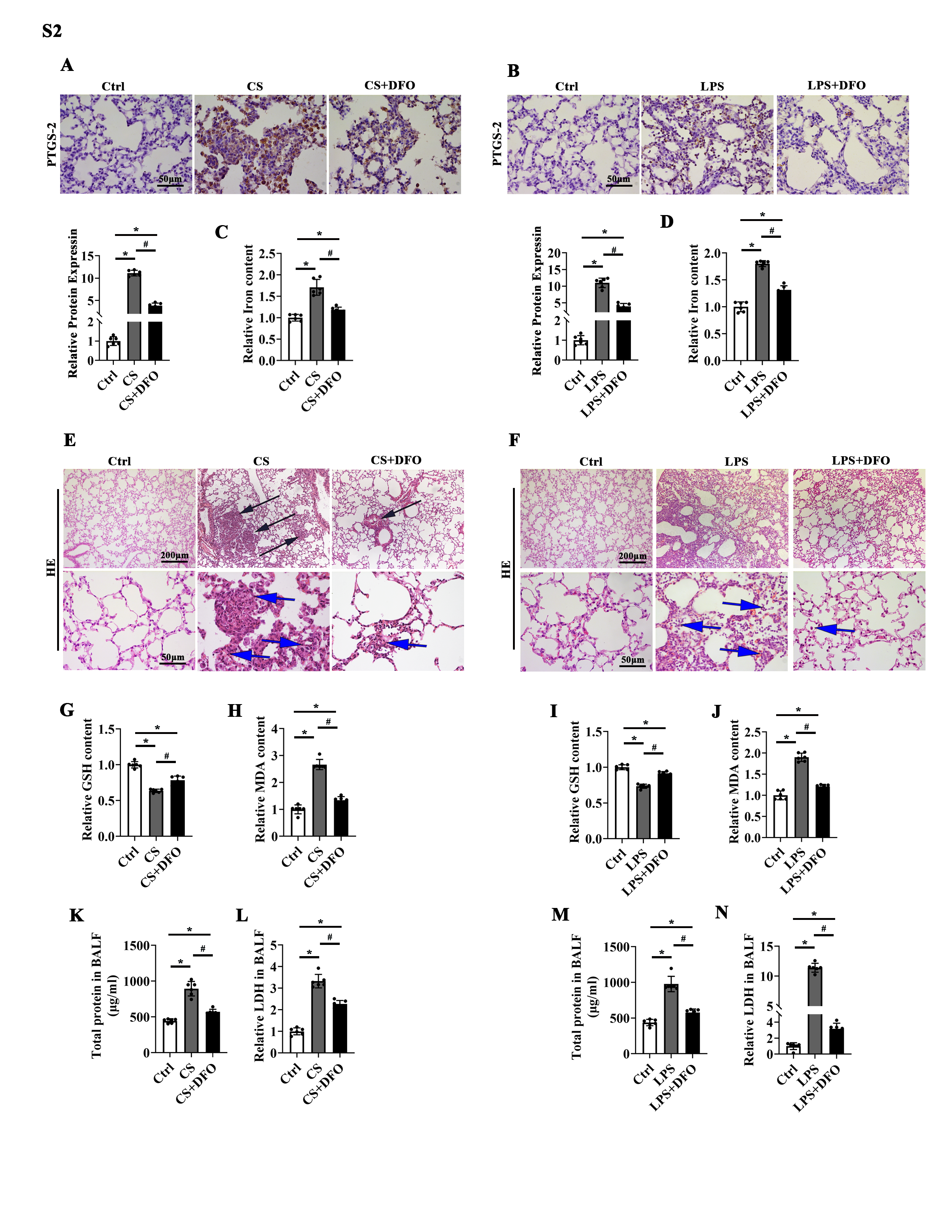

Supplement: Supplementary file 3 — Figure S2 [file 41419_2022_4971_MOESM3_ESM.tif]

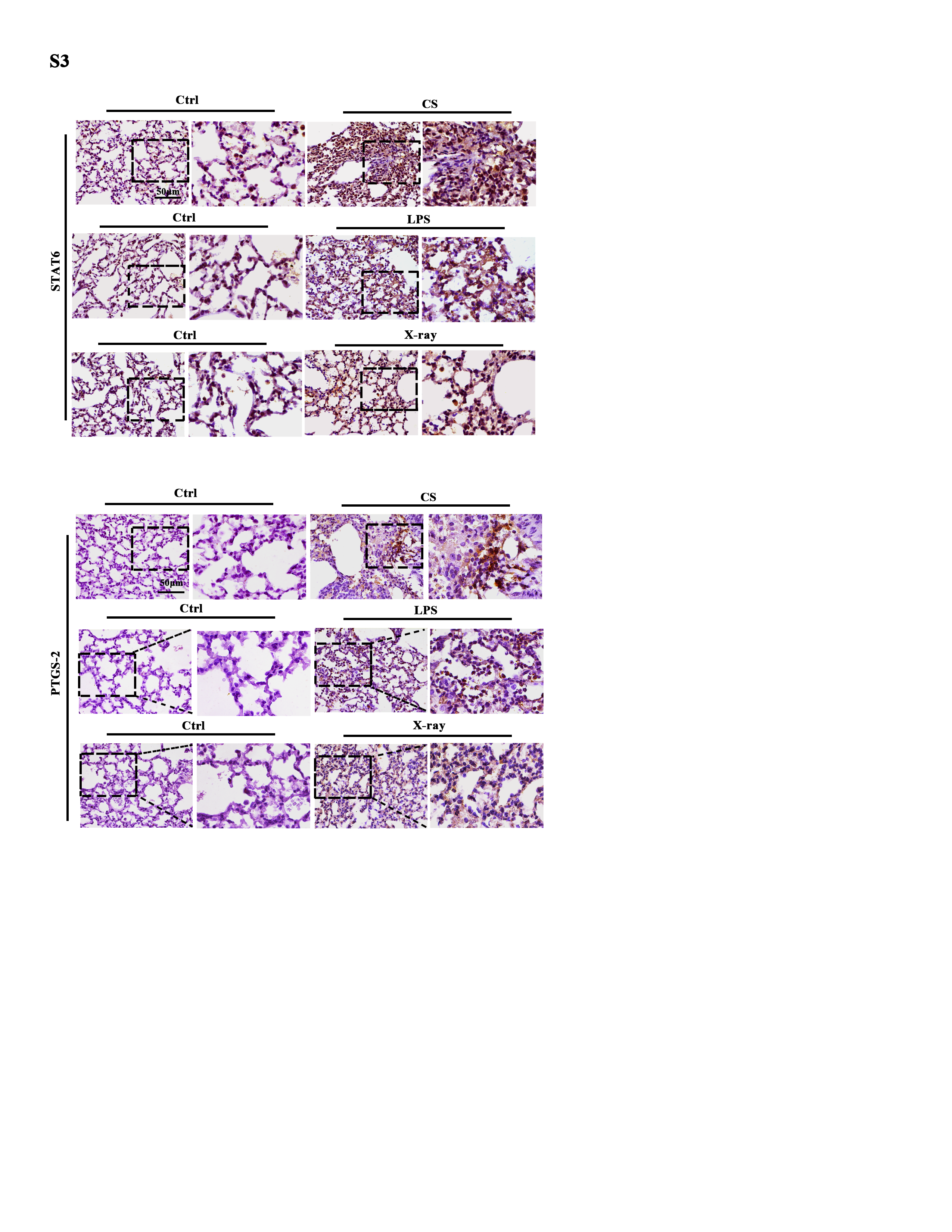

Supplement: Supplementary file 4 — Figure S3 [file 41419_2022_4971_MOESM4_ESM.tif]

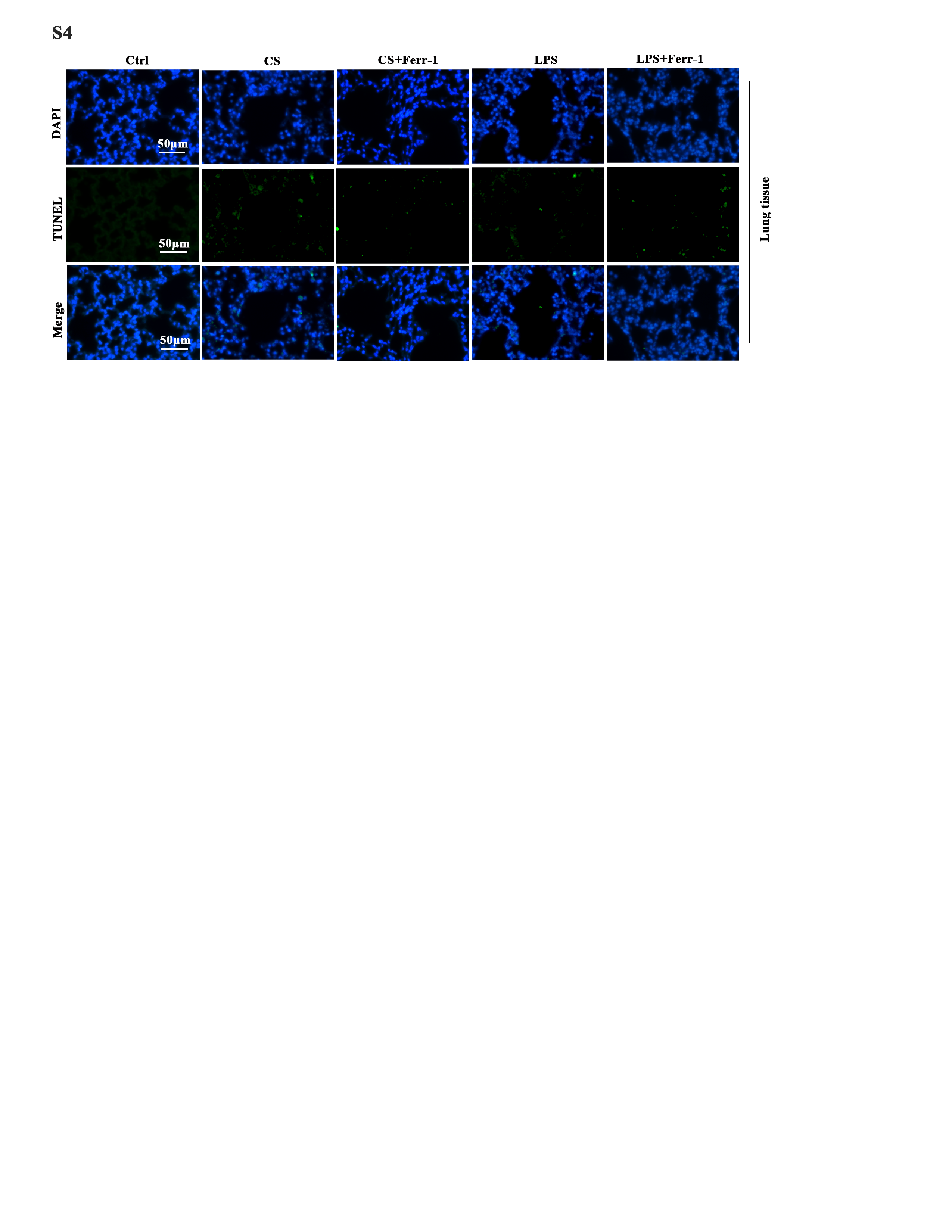

Supplement: Supplementary file 5 — Figure S4 [file 41419_2022_4971_MOESM5_ESM.tif]

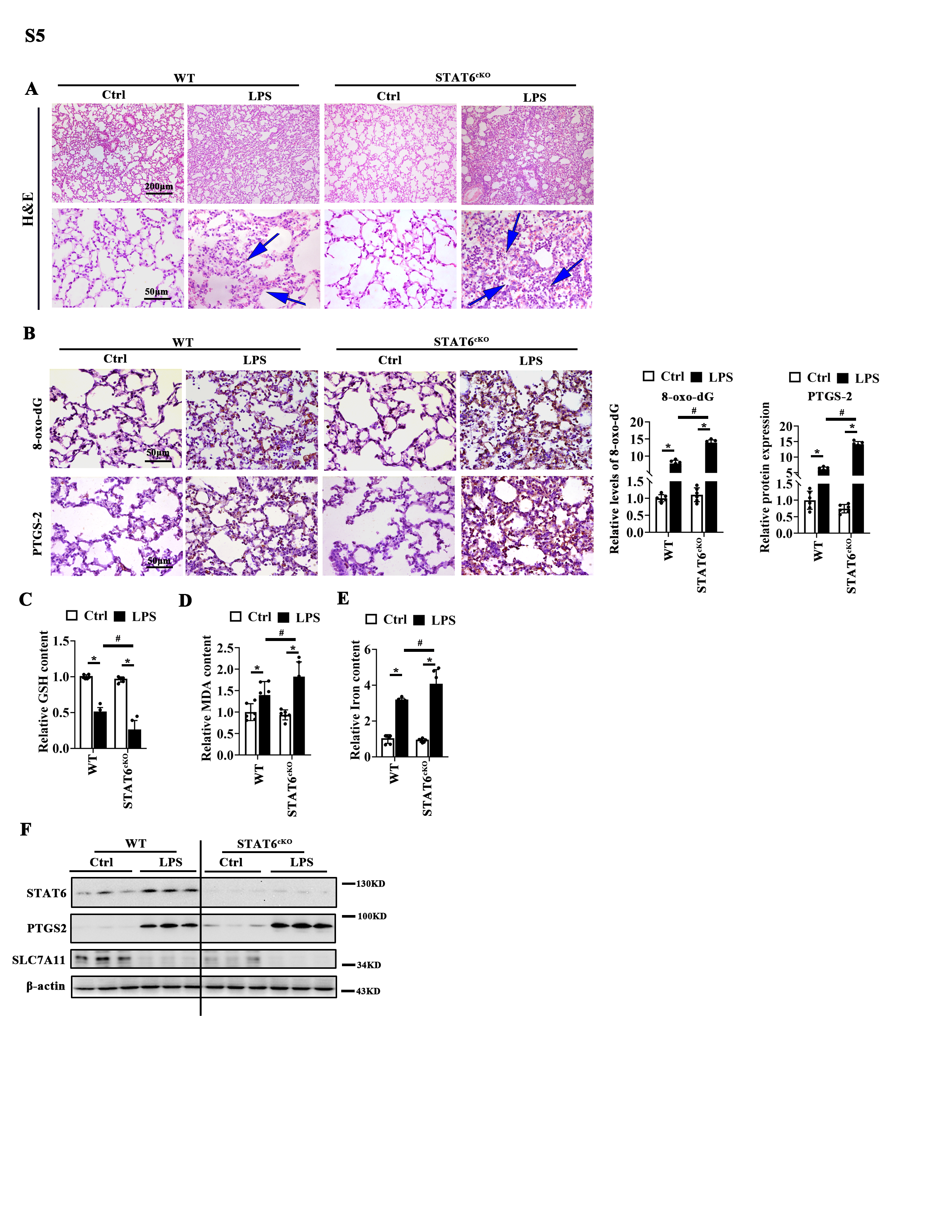

Supplement: Supplementary file 6 — Figure S5 [file 41419_2022_4971_MOESM6_ESM.tif]

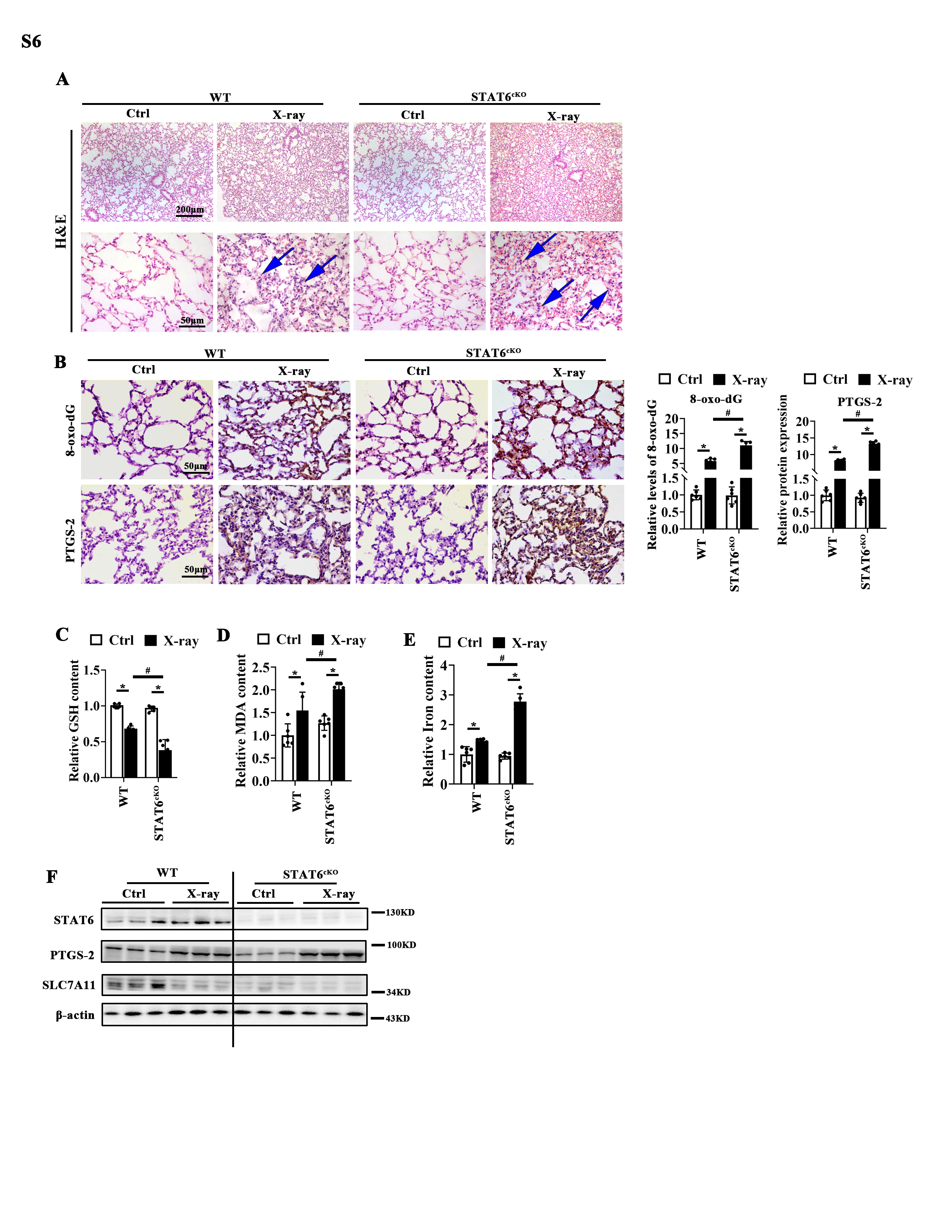

Supplement: Supplementary file 7 — Figure S6 [file 41419_2022_4971_MOESM7_ESM.tif]

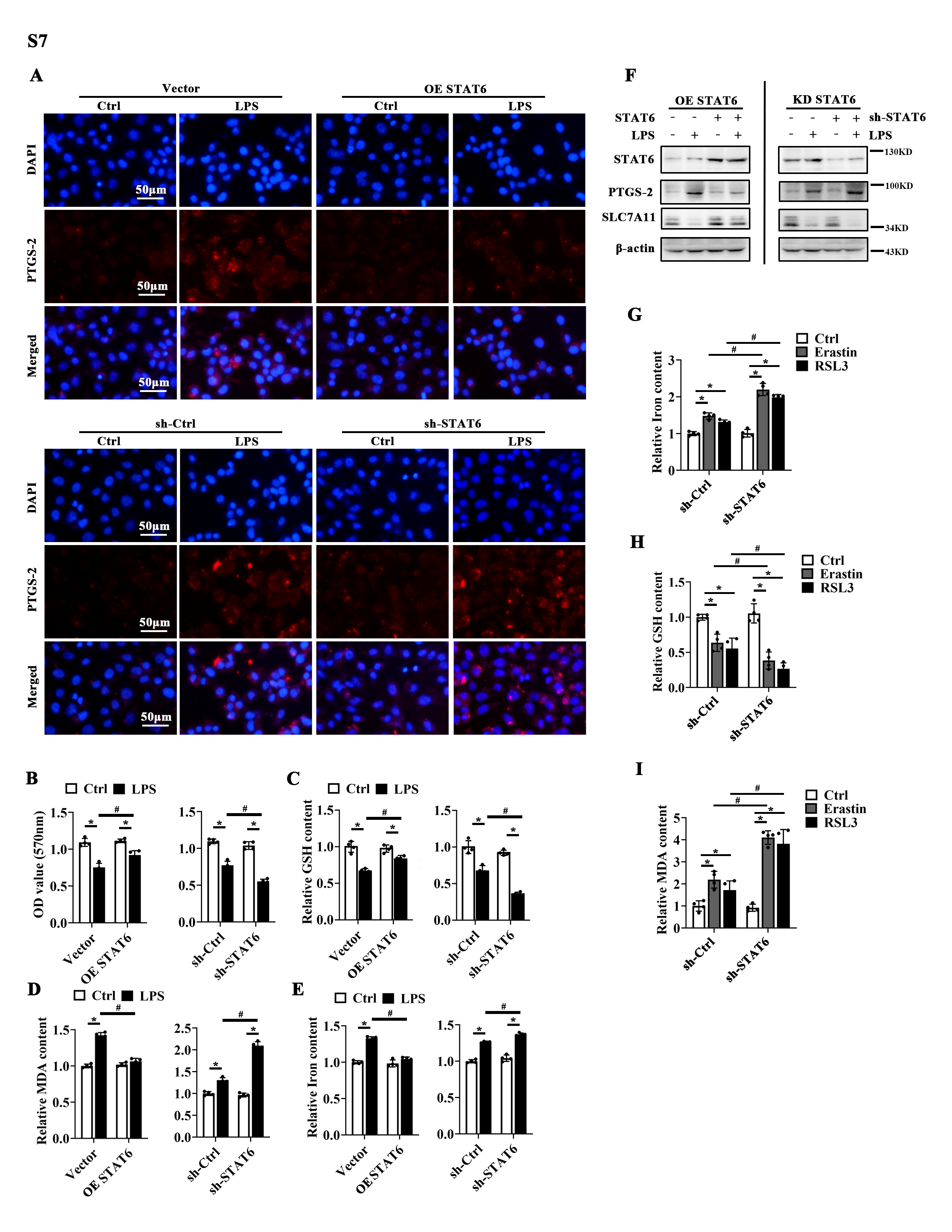

Supplement: Supplementary file 8 — Figure S7 [file 41419_2022_4971_MOESM8_ESM.tif]

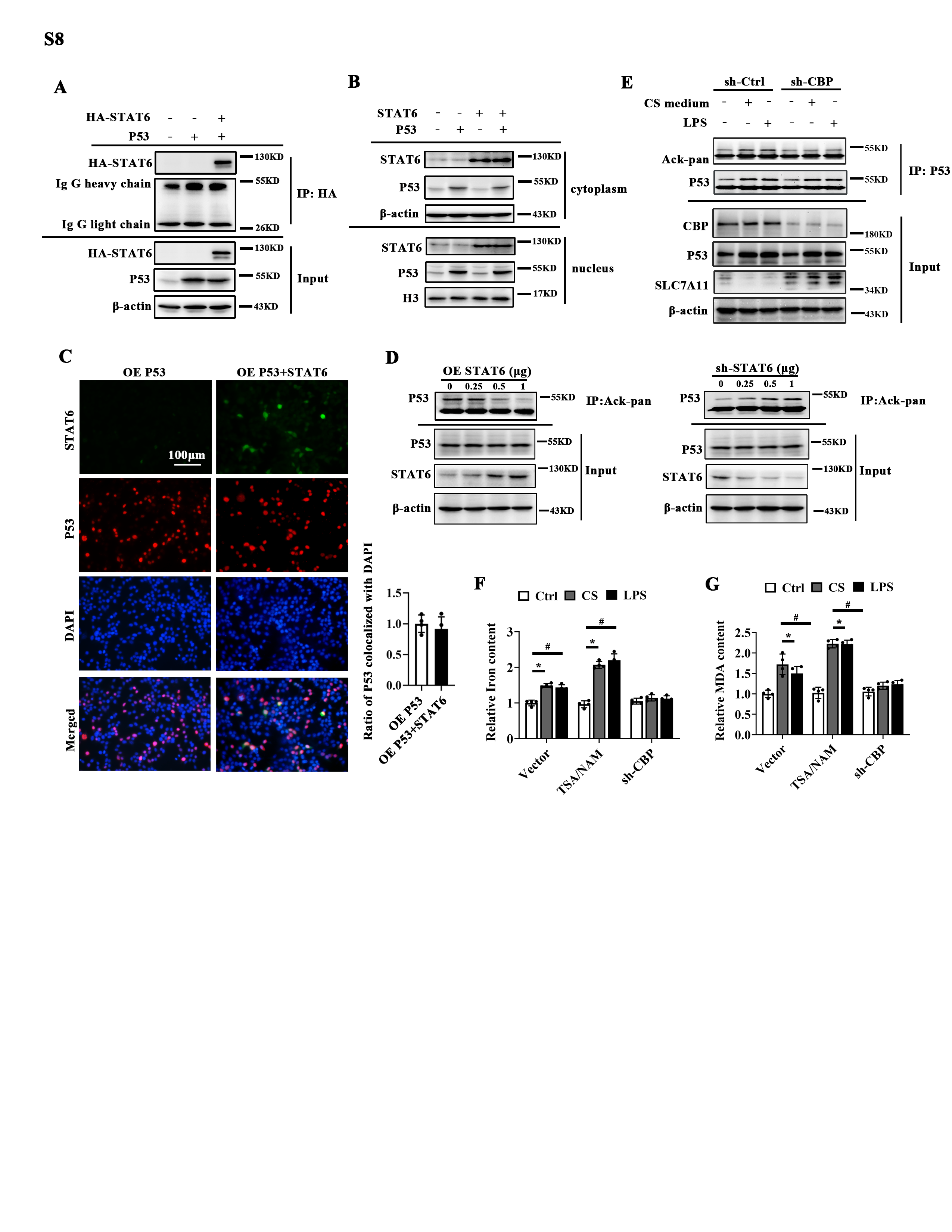

Supplement: Supplementary file 9 — Figure S8 [file 41419_2022_4971_MOESM9_ESM.tif]

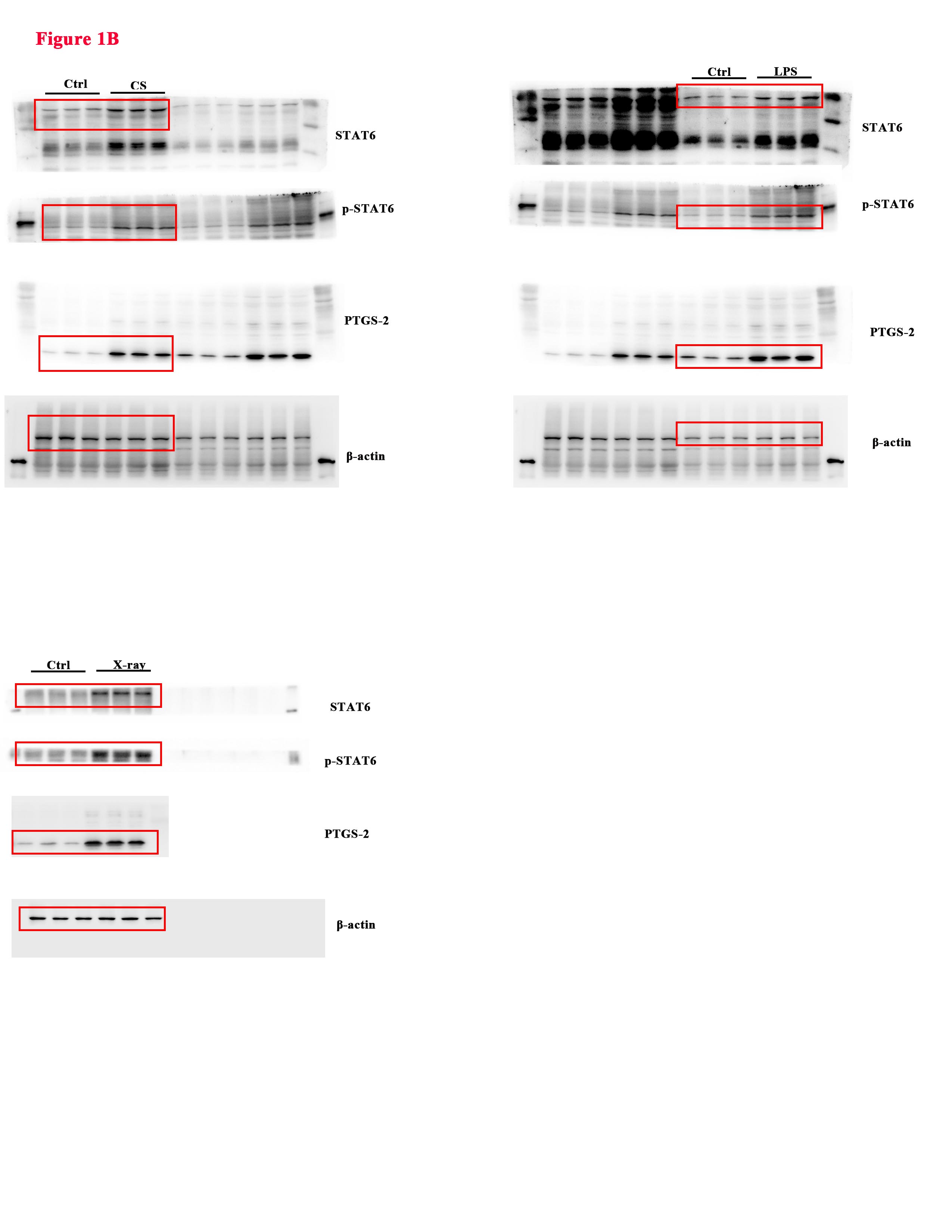


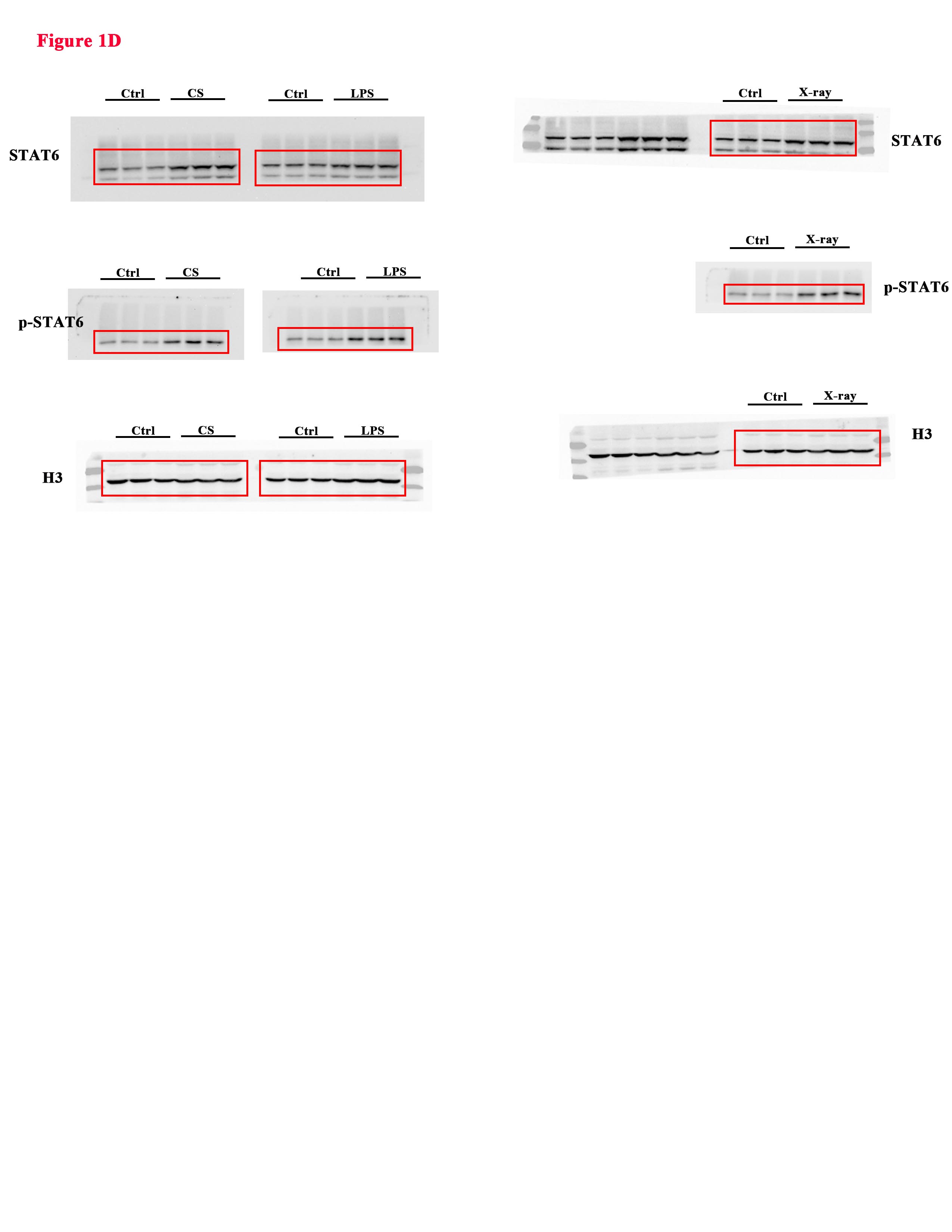

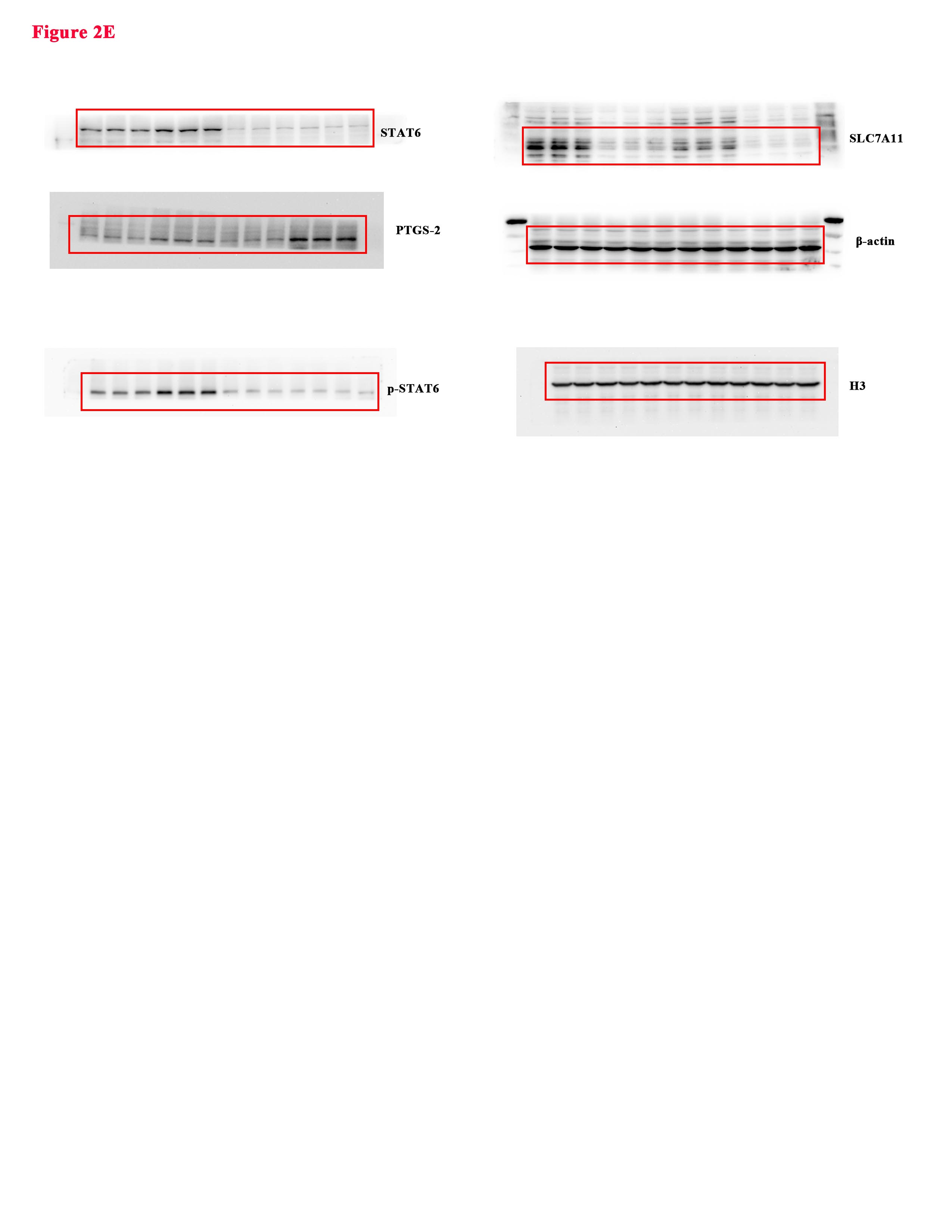

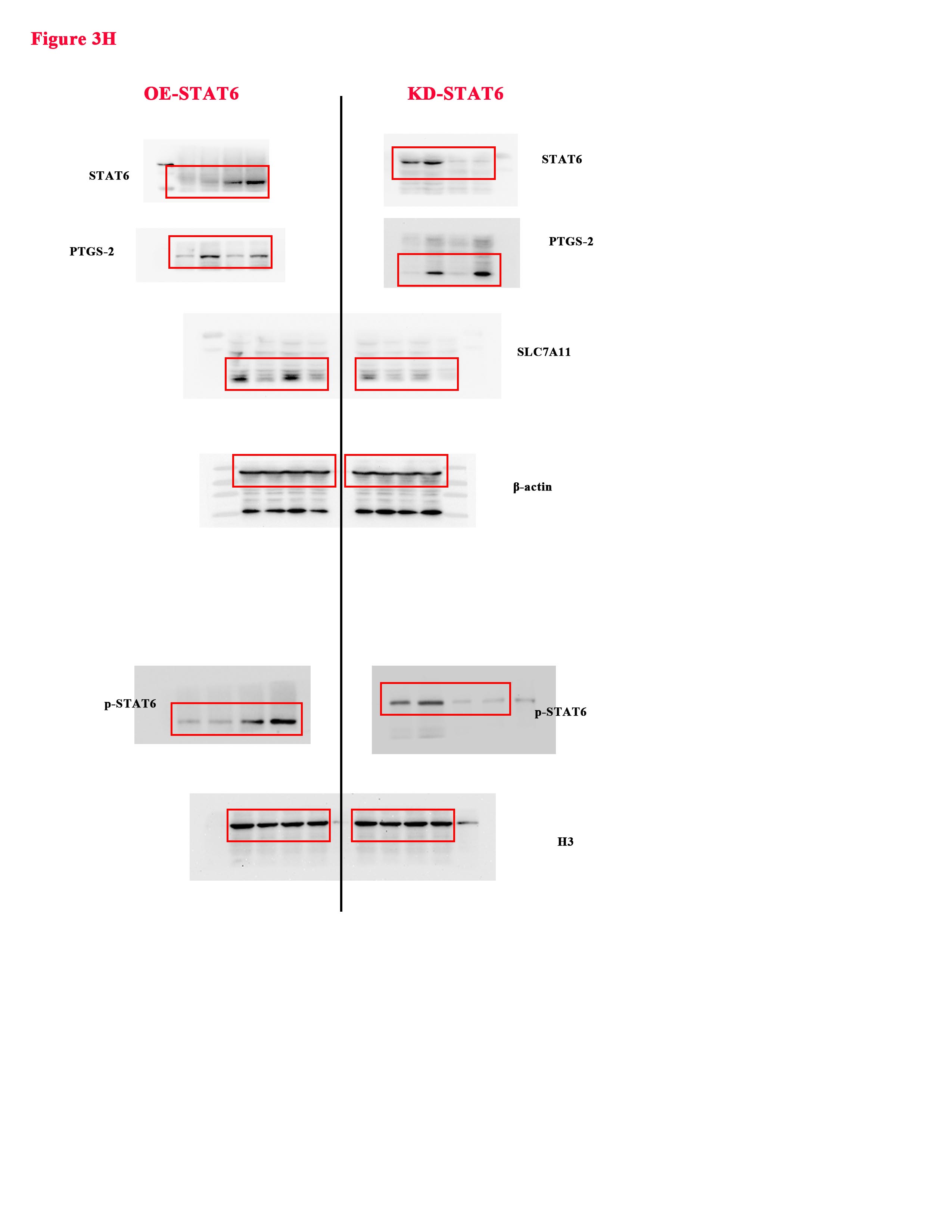


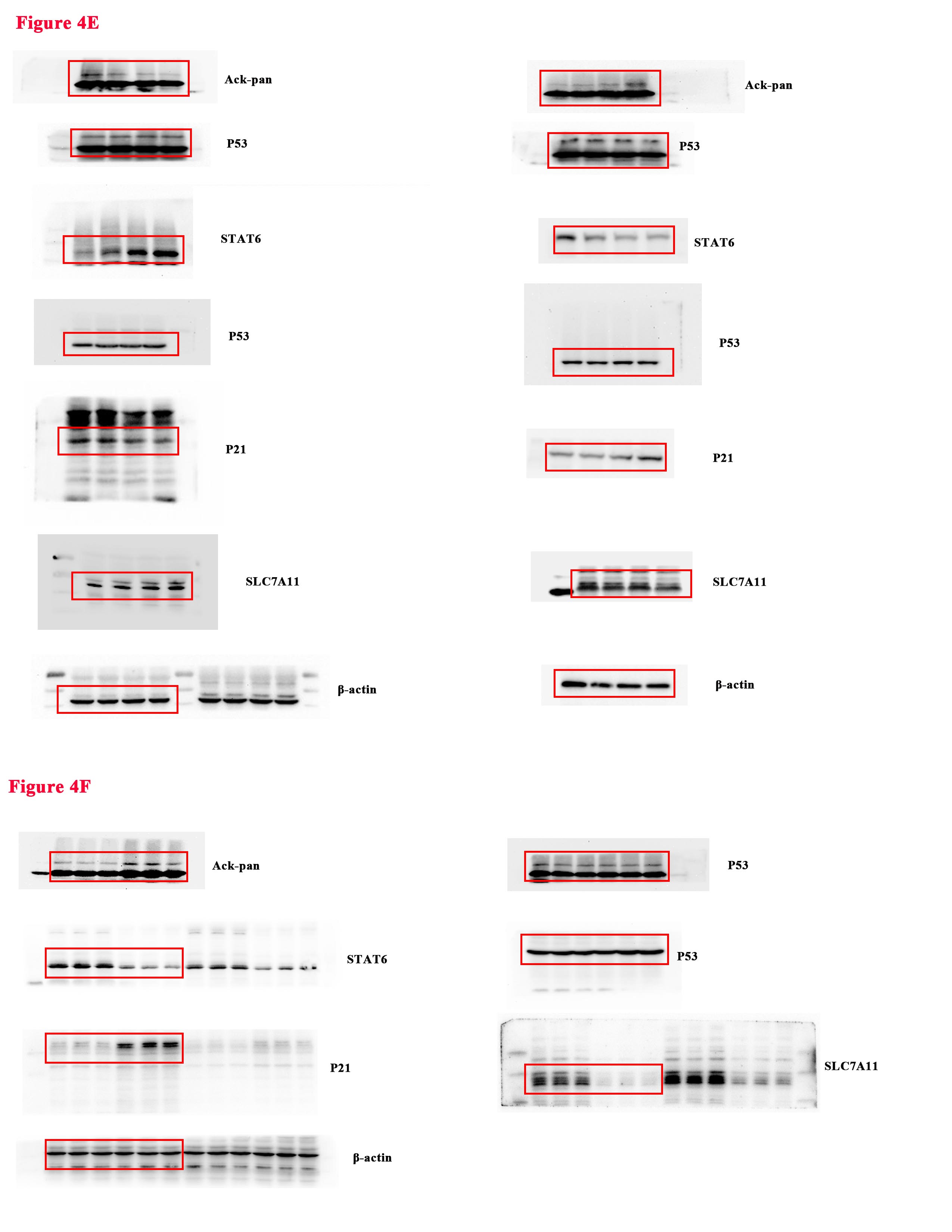


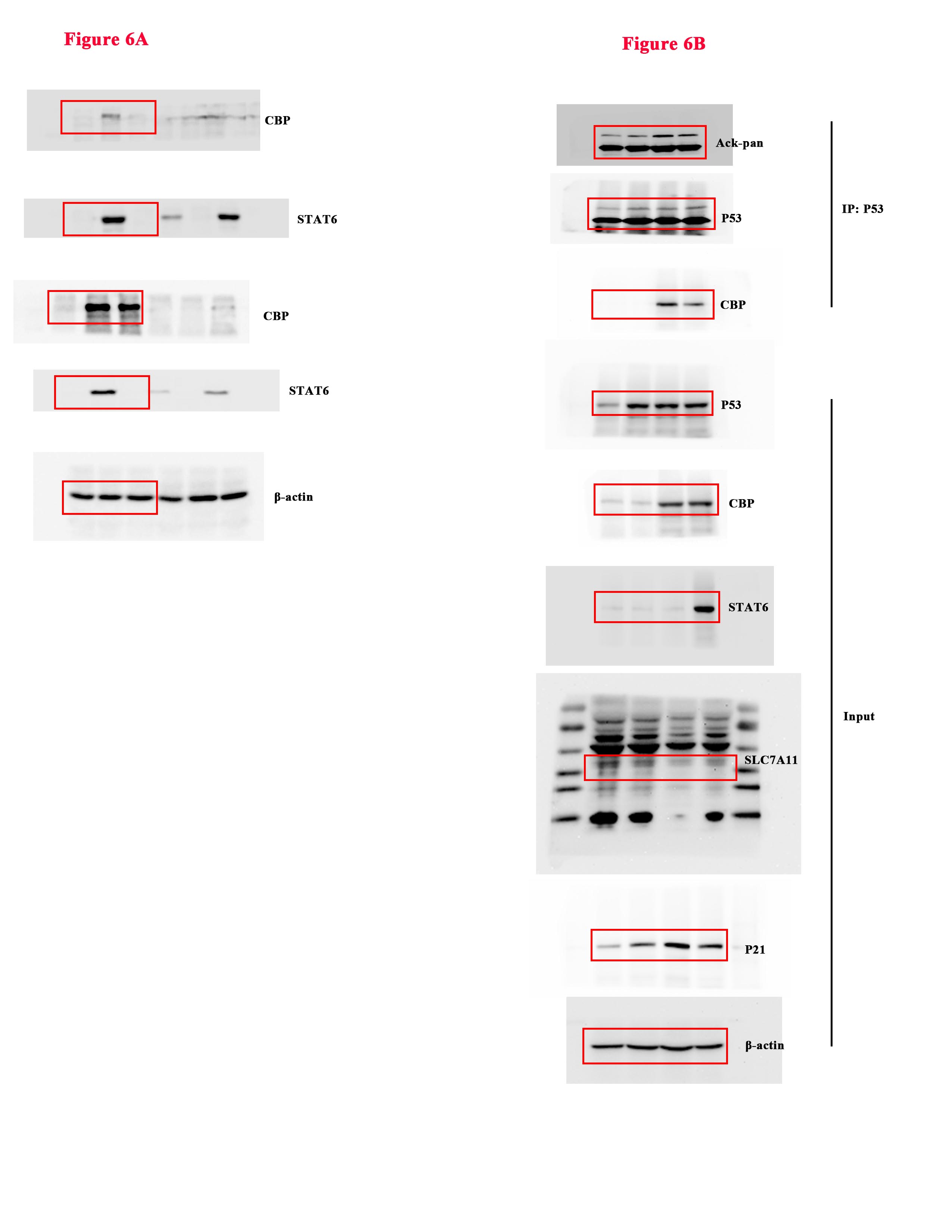


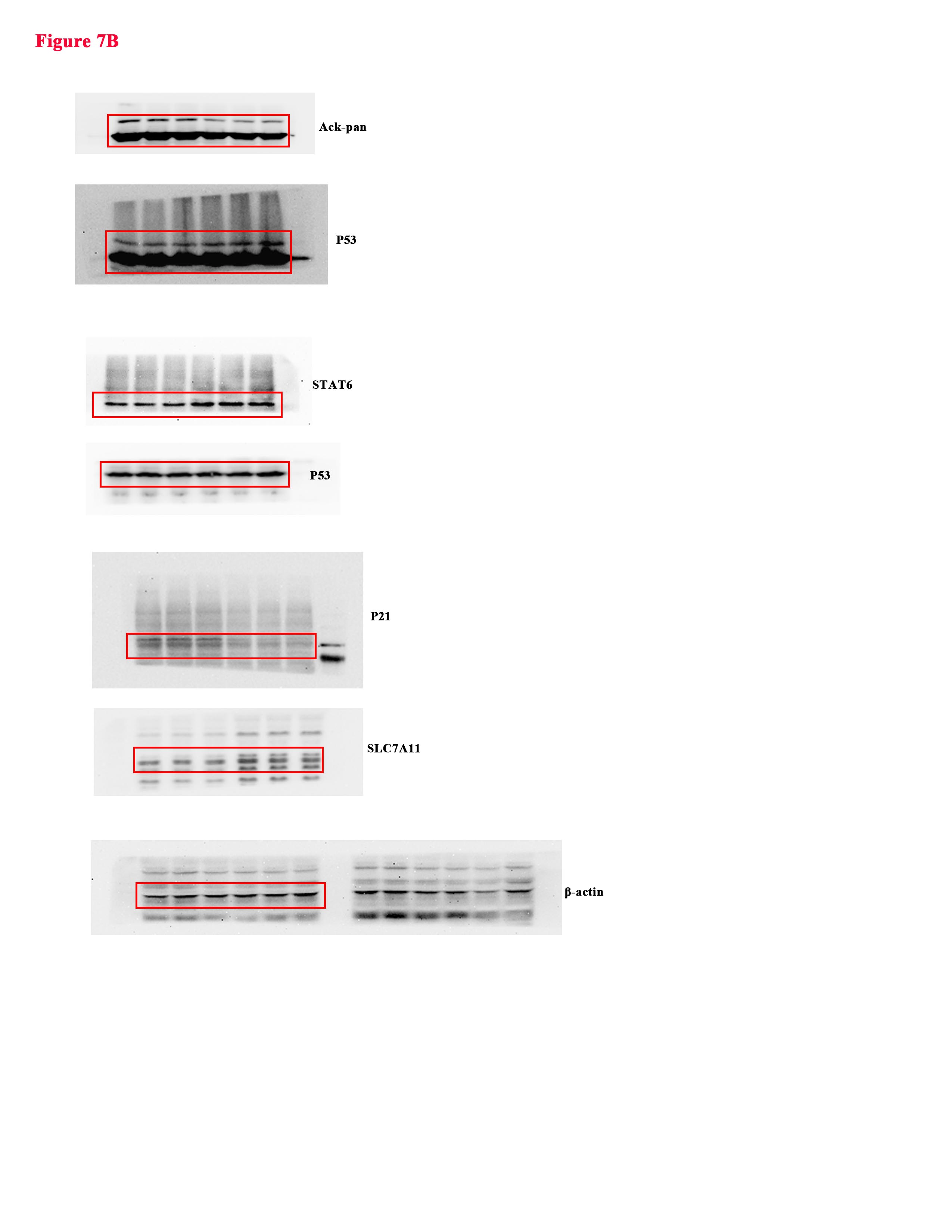


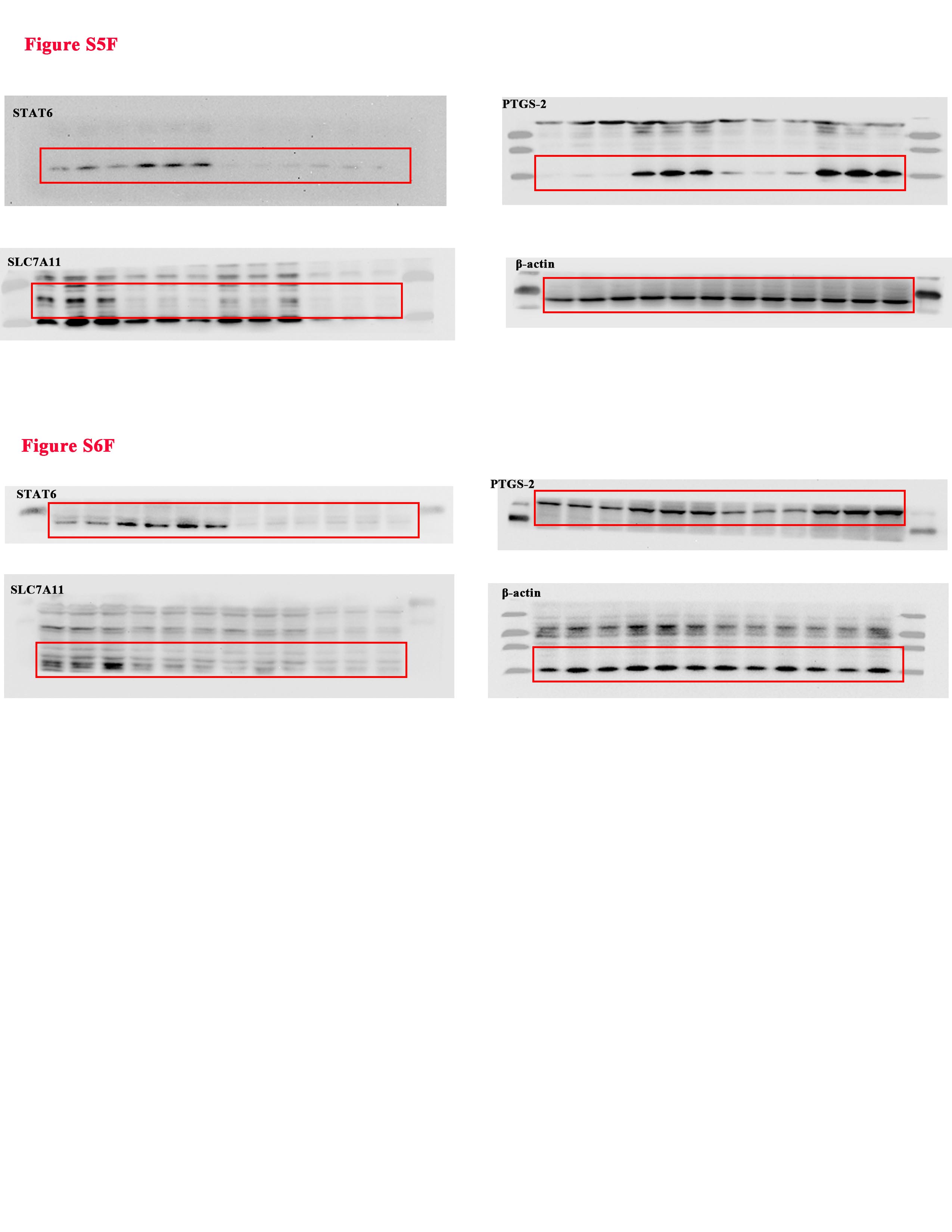


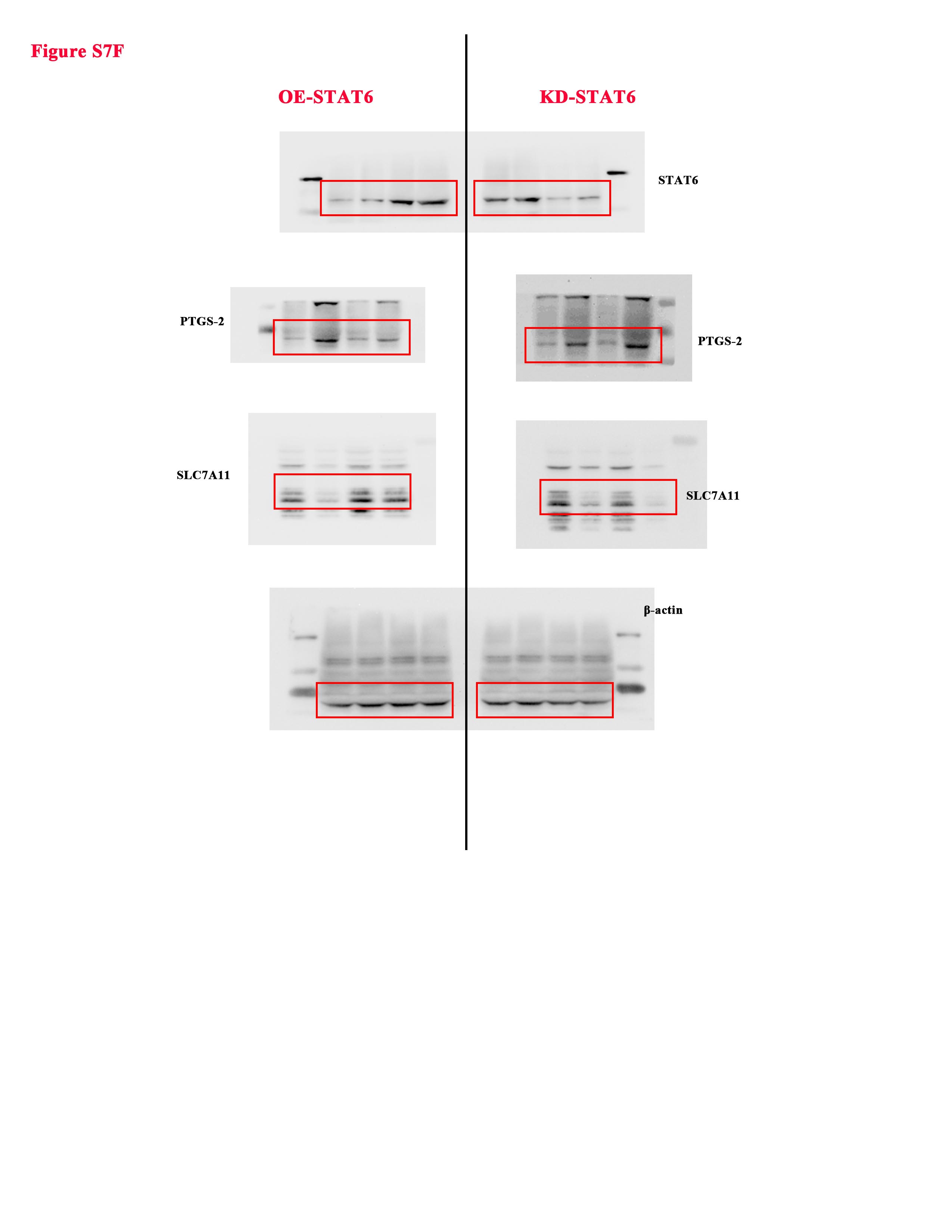


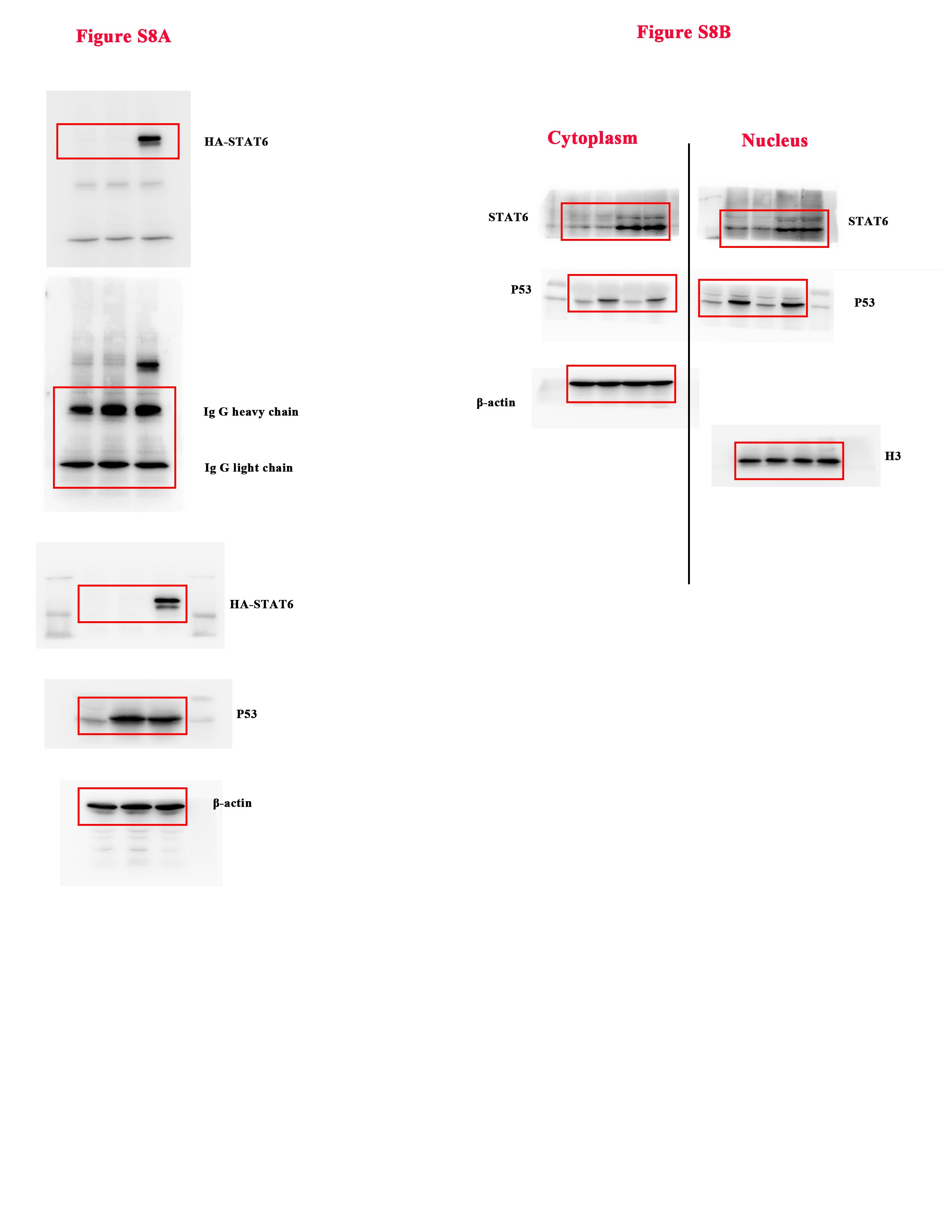


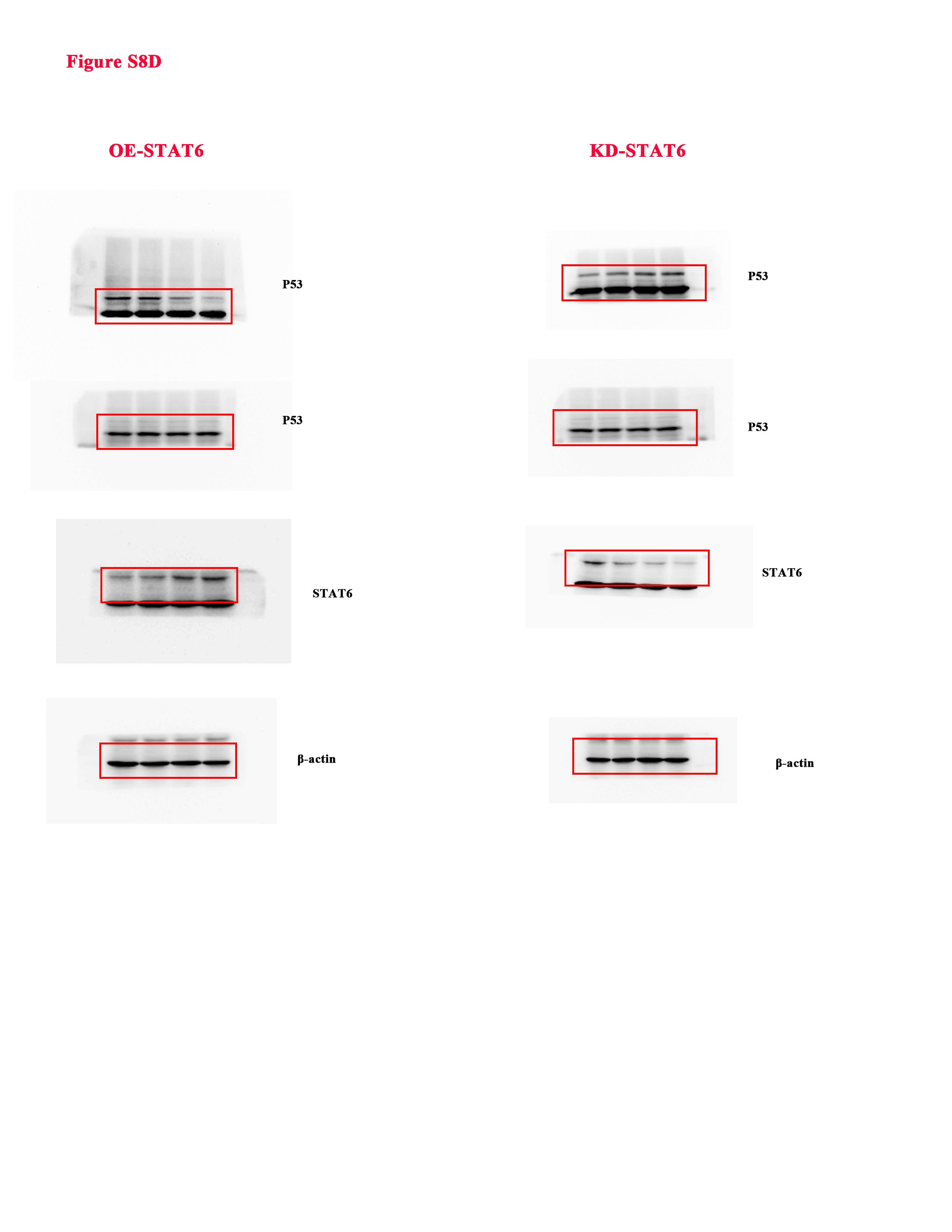

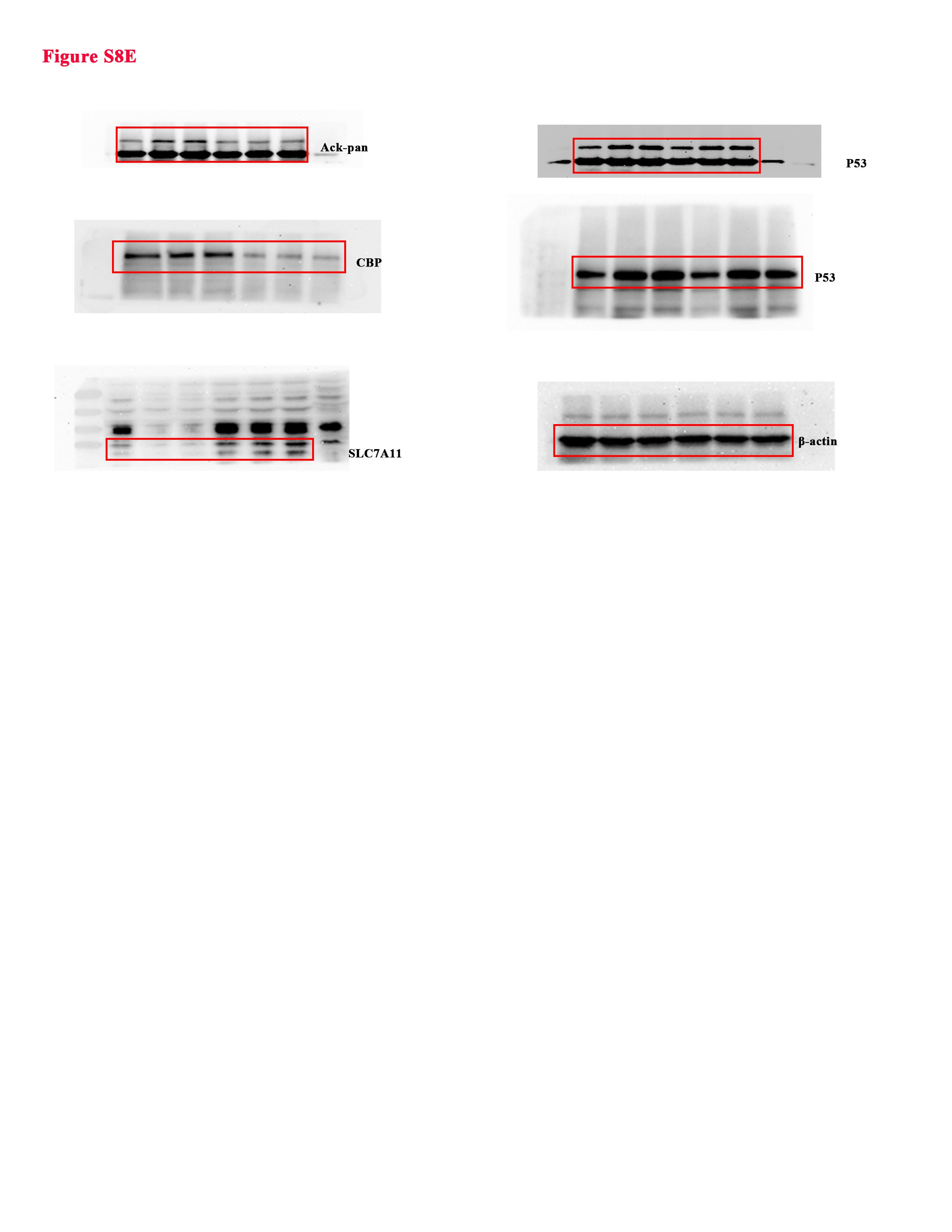

Supplement: Supplementary file 10 — Original Data File [file 41419_2022_4971_MOESM10_ESM.doc]
